# Microbial community composition in the dung of five sympatric European herbivore species

**DOI:** 10.1002/ece3.11071

**Published:** 2024-03-13

**Authors:** Xingzhao Sun, Judith Sitters, Joske Ruytinx, Martin J. Wassen, Harry Olde Venterink

**Affiliations:** ^1^ Research Group WILD Vrije Universiteit Brussel Brussels Belgium; ^2^ B‐WARE Research Centre Nijmegen The Netherlands; ^3^ Research Groups Microbiology and Plant Genetics Vrije Universiteit Brussel Brussels Belgium; ^4^ Environmental Sciences, Copernicus Institute of Sustainable Development Utrecht University Utrecht The Netherlands

**Keywords:** bacterial community, functional prediction, fungal community, herbivore dung, nutrient composition

## Abstract

The dung microbiome is a complex system that is highly influenced by species and diet. This study characterized the dung bacterial and fungal communities of five herbivore species inhabiting the National Park Zuid‐Kennemerland, the Netherlands. The five selected herbivore species were rabbit (*Oryctolagus cuniculus* L.), cow (*Bos taurus* L.), horse (*Equus ferus caballus* L.), fallow deer (*Dama dama* L.), and European bison (*Bison bonasus* L.). We explored the effects of distinct digestive physiology (ruminants vs. non‐ruminants) and diverse dietary preferences on the microbial community composition of herbivore dung. *Firmicutes* and *Bacteroidetes* were dominant bacterial phyla in the dung of all five herbivore species, and *Ascomycota* was the predominant fungal phylum. *Verrucomicrobiota* and *Mucoromycota* were more present in horse dung and Proteobacteria were more abundant in rabbit dung than the three ruminant dung types. There were few significant differences in the microbial community structure among the three ruminant dung types. The alpha and beta diversity of dung microbial communities significantly differed between ruminants and non‐ruminants, especially in bacterial communities. Based on MetaCyc pathways, we found that the primary functions of bacteria in herbivore dung were focused on biosynthesis, various super pathways, and degradation, with a few differences between ruminant and non‐ruminant dung. FUNGuild analysis showed that horse dung had more saprotrophic fungi, while the fungi in fallow deer dung had more symbiotrophic properties, with the fungal functions of bison, cow, and rabbit dung somewhere in between. There was also a correlation between microbial community and nutrient composition of the substrate in herbivore dung. Understanding the dung microbial community composition of these herbivore species can enrich the database of mammalian gut microbiomes for studying the mechanisms of microbial community variation while preparing for exploring a new perspective to study the impact of herbivores on ecosystems through dung deposition.

## 1 INTRODUCTION

Microorganisms are of crucial importance for life on Earth, collaborating, competing, and interacting with other organisms and substrates (Maron et al., 2018; Shu & Huang, 2022; Zinger et al., 2012). In recent decades, gut microecology has been an area of microbial research that increasingly receives attention (Kuziel & Rakoff‐Nahoum, 2022). The mammalian gut microbiomes are assistants in food digestion and energy absorption and decomposers that degrade organic materials back into the nutrient cycling system after entering the external environment as dung (Dearing & Kohl, 2017; Greff et al., 2022).

Studies on the drivers of variation in the mammalian gut or dung microbiota have typically concentrated on species, dietary preferences, and environmental factors. Specifically, in the study of the microbial community of herbivore dung, distinct digestive physiology among species is a primary potential factor causing the differences (Reese & Dunn, 2018). As rumen fermenters, ruminants conduct the degradation of complex cellulose primarily in the anterior part of the digestive system, which requires microbial taxa with specialized metabolic functions to operate in the foregut, unlike hindgut fermenters such as horses (Godoy‐Vitorino et al., 2012; Newbold & Ramos‐Morales, 2020; Stewart et al., 2018). In addition, dietary composition is one determinant of the microecological diversity of the mammalian gut or dung (Kartzinel et al., 2019; Reese & Dunn, 2018). For example, the dung of herbivores consuming high‐fiber plants had a higher diversity of microbial communities than those of carnivores and omnivores (Ley et al., 2008). Likewise, microbial community characteristics in dung differed among herbivores of the same species when distinct subspecies had spatial variation in diet led by geographic factors (Budd et al., 2020). Such variations of the microbial community in the dung due to differences in dietary composition were also significant between captive and wild populations (Gao et al., 2019; Guan et al., 2017; Liu et al., 2021). However, the majority of the data on gut or dung microbial communities of mammalian herbivores comes from captive populations associated directly with the production and livelihood of humans (Boshuizen et al., 2021; Dowd et al., 2008; Durso et al., 2010; Metcalf et al., 2017; Stewart et al., 2018). With specific human needs to be catered to, the diet composition of captive populations is more stabilized and directional than that of wild populations, and the ingestion of plant‐based foods is narrower for captive individuals, which causes the data from captive populations to be somewhat less informative when studying the wild. Such data deficiency of variations in dung microbial communities among wild mammalian herbivore species with diverse dietary preferences and different digestive physiology is detrimental to the theoretical development of microecology and modelings of microbial community variations.

The gut or dung microbiome is mainly composed of bacteria, fungi, and archaea, but most studies have focused on the bacterial community because of the absolute dominance of bacteria (Dougal et al., 2017; Dowd et al., 2008; Durso et al., 2010; Hoffmann et al., 2013; Newbold & Ramos‐Morales, 2020; O'Donnell et al., 2013). Although fungi also have an impact on maintaining animal health and supporting metabolic functions, they have not yet gained adequate understanding (Newbold & Ramos‐Morales, 2020; Sokol et al., 2017). For example, anaerobic fungi in the ruminant gut can degrade high‐fiber foods, especially when the ruminant does not have high‐quality foraging (Huws et al., 2018; Krause et al., 2013). Therefore, it is necessary to combine the fungal and bacterial flora to comprehensively study the microbial community composition of the herbivore dung.

In addition to assisting herbivores in digesting food and converting energy, the dung microbiota is also involved in the degradation and cycling of nutrients in the ecosystem. For example, as resident dominant bacteria in the dung microbial community, *Bacteroidota* and *Firmicutes* are capable of N mineralization (Burns & Qin, 2023). Furthermore, the process of nutrient return to the ecosystem through the decomposition of the substrate is also associated with the dung C:N:P stoichiometric characteristics (Anderson & Coe, 1974; Ouédraogo et al., 2004). The rates of dung deposition, decomposition, and nutrient return are affected by the nutrient composition of dung substrates (Sitters et al., 2014). Therefore, it is likely that the diversity of dung microbial composition is correlated with dung nutrients, but additional studies are needed to investigate this.

The objectives of this study were to reveal the microbial community composition, including bacteria and fungi, and predict the functional characteristics of the microbial communities in the dung of five mammalian herbivore species (European bison, cow, fallow deer, horse, rabbit), varying in body size, digestive system, and diet, which sympatrically inhabit European herbaceous and shrubby habitat near coastal dunes. We also aimed to examine relationships among bacterial and fungal communities and between nutrients (C, N, P concentrations and their ratios) and microbial community characteristics in the dung of these five herbivore species. We hypothesized that the dung microbial community composition and its predicted function groups of the five sympatric herbivore species would be significantly different due to their distinct digestive physiology (ruminants vs. non‐ruminants) and dietary preferences and that there would also be some correlations between these differences in microbial community structure and the dung nutrients. This study aims to enrich the dung microbiome database and to pre‐prepare for a potentially new perspective for studying the impact of herbivore dung on ecosystems.

## 2 MATERIALS AND METHODS

### 2.1 Sample collection

**FIGURE 1:**
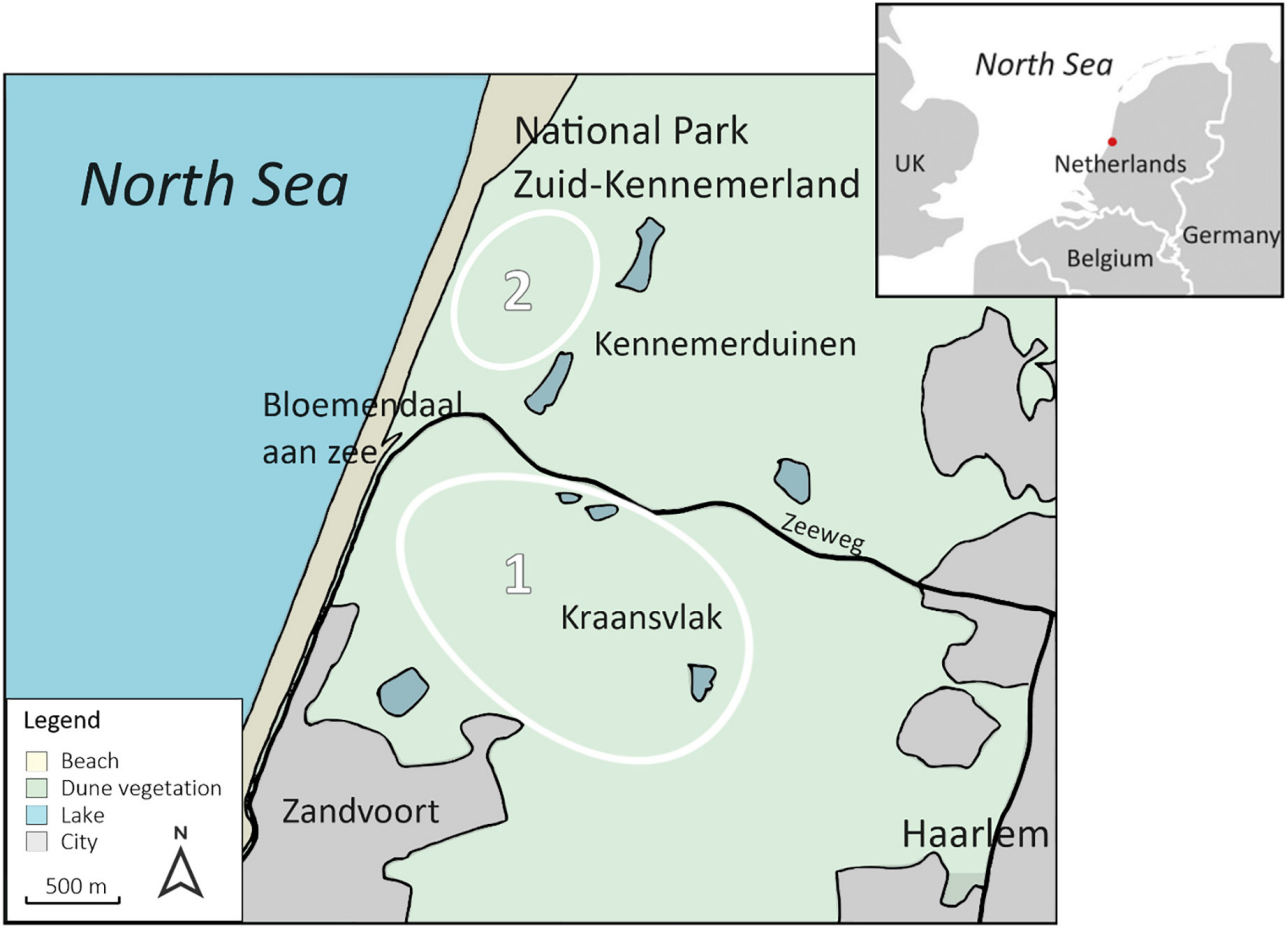
Map of the dung sampling area, the National Park Zuid‐Kennemerland in the Netherlands. The dung samples of European bison, cow, horse, and fallow deer as well as part of rabbit dung were collected in “Het Kraansvlak” (indicated with white circle 1), rabbit dung was mostly collected in the area northwest of Kraansvlak closer to the sea (indicated with white circle 2). Six replicate samples were collected from different individuals per species and at different locations in these areas.

Dung samples of rabbit (*Oryctolagus cuniculus* L.), cow (*Bos taurus* L.), horse (*Equus ferus caballus* L.), fallow deer (*Dama dama* L.), and European bison (*Bison bonasus* L.)were collected in the National Park Zuid‐Kennemerland, the Netherlands (52.3961° N, 4.5921° E) on February 25th, 2020. We chose these five herbivore species because they cover a range in body mass, include different digestive systems (ruminant vs. non‐ruminant), cover different diets (grazer, mixed feeder), and occur in the same National Park covering a variety of habitats (dunes, dune slacks, grasslands, shrubs, woodland). The first three species are very common in nature reserves in Europe, the other two species increased the variation in herbivore types. The National Park Zuid‐Kennemerland is a heterogeneous coastal dune with a diverse landscape of deciduous and pine forest, buckthorn shrubland, open or sparse meadow, and abandoned arable (Cromsigt et al., 2007). A number of European bison, cow, and horse have been introduced since 2008 to counteract the invasion of woody plants, and the area is also home to numerous free‐roaming deer and rabbits (Kerley et al., 2012; Naundrup & Svenning, 2015; Valdés‐Correcher et al., 2018; Van Strien et al., 2011). We collected six replicate dung samples per herbivore species, with a focus on collection from different individuals. Dung from horse, cow, and European bison was mainly collected directly after excretion. The animals were observed for a while to make sure different individuals were sampled. The six replicate dung samples from rabbit and fallow deer were collected at six different locations in the sampling area, assuming they were from different individuals. Rabbit and fallow deer have many individuals in the Park. We only sampled fresh dung pallets. The sample‐collecting map of the National Park Zuid‐Kennemerland and the dietary preferences of the five herbivore species are detailed in Figure 1 and Table 1. All fresh herbivore dung samples were sealed and brought back to the Vrije Universiteit Brussel (VUB), stored in the dark at 4°C until subsequent processing in the laboratory.

**TABLE 1:** Diet characteristics of five herbivore species inhabiting European coastal dunes.

Herbivore species	Digestive system	Diet preference	Reference
European bison	Ruminant	80% Grass, 20% woody plants (barks)	Cromsigt et al. (2017); Valdés‐Correcher et al. (2018)
Cow	Ruminant	80% Grass, 20% woody plants (barks, branches)	Cromsigt et al. (2017); Valdés‐Correcher et al. (2018)
Fallow deer	Ruminant	Grass, herbs, and woody plants (barks, twigs, buds)	Ramirez et al. (2021); Saint‐Andrieux et al. (2009)
Horse	Monogastric	Grass, woody plants (barks, twigs)	Kuiters et al. (2006); Rupprecht et al. (2016)
Rabbit	Monogastric/Coprophagous	Herbs, grasses, and some woody vegetation (saplings, roots, shoots)	Ebino et al. (1993); Van Strien et al. (2011)

### 2.2 Determination of nutrient concentrations

For microbial environmental factors analysis, we measured the moisture (%) and total carbon (C), nitrogen (N), and phosphorus (P) concentrations of the dung of the five herbivore species (4 replicates of different individuals of each herbivore species, *n* = 4). The fresh herbivore dung was placed in the oven at 70°C for 72 h for drying to measure the moisture and then ground for the nutrient measurements. Total C concentrations were analyzed with a dry combustion analyzer (LECO CNS‐2000). Total N concentrations were measured on an elemental analyzer (Thermo EA Flash 1112). Total P concentrations were analyzed using a modification of the combustion and hot HCl extraction procedure (Andersen, 1976; Johengen, 1997).

### 2.3 DNA extraction and sequencing

DNA extraction from the dung of the five herbivore species (*n* = 6, six replicates from different individuals per herbivore species) was performed using PowerSoil DNA Isolation Kit (MO BIO Laboratiories, Inc., USA) after which it was stored at −20°C and then sent to the Novogene (UK) Company Limited for high‐throughput sequencing analysis. The V4 region of the bacterial 16S rRNA gene was amplified by 515F/806R primers (Caporaso et al., 2011). The ITS1 region of the fungal internal transcribed spacer (ITS) gene was amplified by ITS5‐1737F/ITS2‐2043R primers (White et al., 1990). Sequencing analysis was performed on the Illumina NovaSeq PE250 sequencing platform. The raw sequencing data of bacteria and fungi obtained from this research project were deposited in the NCBI Sequence Read Archive under the accession number PRJNA853949.

### 2.4 Bioinformatic analysis

Bioinformatics analysis of raw bacterial and fungal sequencing data was mainly performed on QIIME2. The sequencing data were subjected to data quality control by FLASH software and QIIME2 (Bolyen et al., 2019; Magoč & Salzberg, 2011), and then ASVs (Amplicon Sequence Variants) files with species annotations were obtained by applying the DADA2 method and QIIME2's classify‐sklearn algorithm (Bokulich et al., 2018; Callahan et al., 2016). QIIME2 was also used to calculate the alpha diversity indices of bacteria and fungi, such as the Chao1 richness index, Shannon–Wiener diversity index, Pielou evenness index, and Simpson index, and non‐metric multi‐dimensional scaling analysis (NMDS) for beta diversity. The functional prediction of bacteria was carried out in the PICRUSt2 according to 16S sequencing data based on the MetaCyc metabolic pathways database (Caspi et al., 2020; Douglas et al., 2020). The FUNGuild annotation tool was used to parse fungal functional trophic mode and ecological guilds (Nguyen et al., 2016).

### 2.5 Statistical analysis

Differences among species in dung nutrients were determined using a one‐way ANOVA and Scheffe test in the agricolae package (de Mendiburu, 2021), whereas statistical differences of alpha diversity indices and relative abundance of microbial communities and their functional communities calculated by the sequencing data were performed using Kruskal–Wallis test followed by Dunn's multiple comparisons test, which were conducted by the pgirmess, multcomp, and multcompView packages in R (Giraudoux, 2022). Correlations between microbial community characteristics and environmental factors (herbivore dung nutrients) were evaluated by Pearson correlation analysis, Mantel test, and redundancy analysis (RDA). The RDA is performed and plotted by Canoco 5. Pearson correlation analysis and the Mantel test were evaluated and plotted by the corrplot and ggcor packages in R (Huang et al., 2022; Wei et al., 2021). Correlation‐based ecological network analysis was conducted to examine the associations between bacteria and fungi at the class level in the dung of the five herbivore species. The analysis employed the psych, Hmisc, and igraph packages in R (Csárdi et al., 2024; Harrell Jr., 2023; Revelle, 2023). Pairwise Spearman's rank correlations (*r*) were calculated for the relative abundance (>0.1%) of microbial classes, with a focus on robust correlations (|*r*| > .6) that were statistically significant (*p*‐values < .05). The network was visualized, and topological parameters were derived using the interactive platform Gephi version 0.10.1.

## 3 RESULTS

### 3.1 Herbivore dung nutrients and moisture

The dung of the five herbivore species varied in moisture content, as well as C, N, and P concentrations and ratios among these elements (Table 2). The dung of European bison and cow had a higher moisture content than that of fallow deer and rabbit. Rabbit dung was relatively rich in N, but poor in C and P, resulting in relatively low C:N but high N:P ratios. Fallow deer dung was relatively P‐rich. The European bison, cow, and horse dung all had relatively high C:N and relatively low N:P ratios.

**TABLE 2:** Moisture and nutrient concentrations in the dung of five herbivore species collected in the dune area of the National Park Zuid Kennemerland, the Netherlands in February 2020.

Herbivore species	Moisture (%)	TN (g kg^−1^)	TC (g kg^−1^)	TP (g kg^−1^)	C:N	N:P
Bison	81.15 ± 0.94^a^	0.14 ± 0.00^bc^	4.82 ± 0.05^ab^	0.02 ± 0.00^ab^	34.82 ± 0.87^b^	6.21 ± 0.22^b^
Cow	81.90 ± 1.43^a^	0.13 ± 0.00^bc^	5.16 ± 0.03^a^	0.02 ± 0.00^b^	38.84 ± 0.46^ab^	6.97 ± 0.19^b^
Fallow Deer	69.08 ± 0.95^c^	0.16 ± 0.01^b^	4.46 ± 0.09^cd^	0.03 ± 0.00^a^	28.81 ± 0.90^c^	5.12 ± 0.42^b^
Horse	76.15 ± 0.93^ab^	0.12 ± 0.00^c^	4.78 ± 0.04^bc^	0.02 ± 0.00^ab^	39.39 ± 0.75^a^	5.14 ± 0.40^b^
Rabbit	70.28 ± 1.22^bc^	0.18 ± 0.00^a^	4.21 ± 0.05^d^	0.01 ± 0.00^b^	23.21 ± 0.27^d^	12.87 ± 1.59^a^

*Note*: Mean ± Standard error of the mean (SEM) (*n* = 4); Different superscript letters indicate significant differences between species using one‐way ANOVA followed by Scheffe test (*p* < .05).

### 3.2 Alpha and beta‐diversity of the microbiota

**FIGURE 2:**
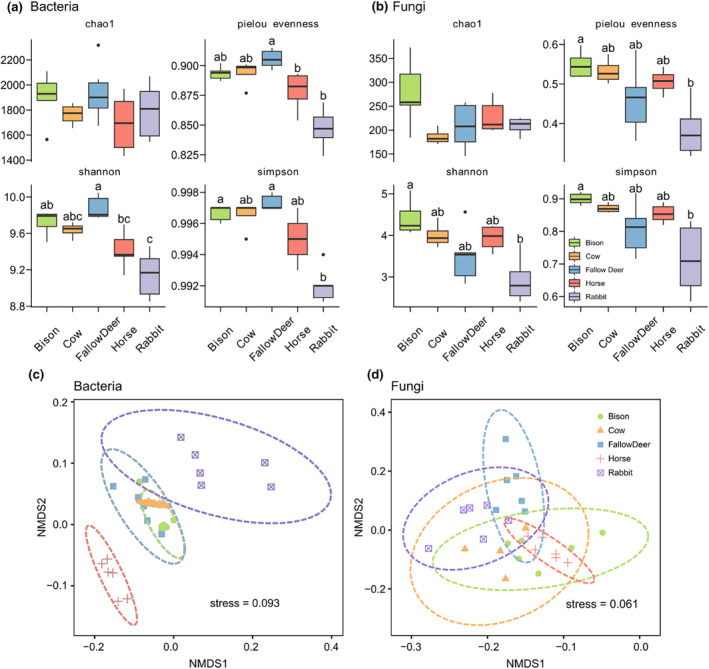
The α‐diversity indicators chao1 index, Shannon–Wiener index, Pielou evenness index, and Simpson index for bacteria (a) and fungi (b) in dung of five herbivore species, as well as non‐metric dimensional scaling (NMDS) with weighted Unfrac distance on ASVs level of the β‐diversity for bacteria (c) and fungi (d). Different letters indicate significant differences at the *p* < .05 level (*n* = 6) of the one‐way Kruskal–Wallis test. The exact *p*‐values of all Kruskal–Wallis tests are listed in Table S1.

Based on the observed ASVs, there was no significant difference in the total number of ASVs including bacteria and fungi, and the proportion of bacteria in the dung microbiota of the five herbivore species. The total ASVs in the dung of each herbivore species averaged about 2000 and bacterial ASVs accounted for about 90% of the microbiota. Yet, alpha metrics describing the diversity of bacteria and fungi differed significantly between herbivore species (Figure 2). The alpha diversity (Shannon–Wiener, Simpson, Pielou evenness) of bacteria was relatively high in fallow deer and low in horse and rabbit (Figure 2a). Fungal diversity was higher in the dung of European bison and lower in that of rabbit (Shannon–Wiener, Simpson, Pielou evenness) (Figure 2b). The beta diversity analysis of bacteria in dung using NMDS resulted in three distinct groups: one horse, two rabbits, and three ruminant herbivore species (Europena bison, cow, and fallow deer) (Figure 2c). There was no clear distinction between beta diversity of fungi in the dung of the five herbivore species (Figure 2d).

### 3.3 Microbial community composition

**FIGURE 3:**
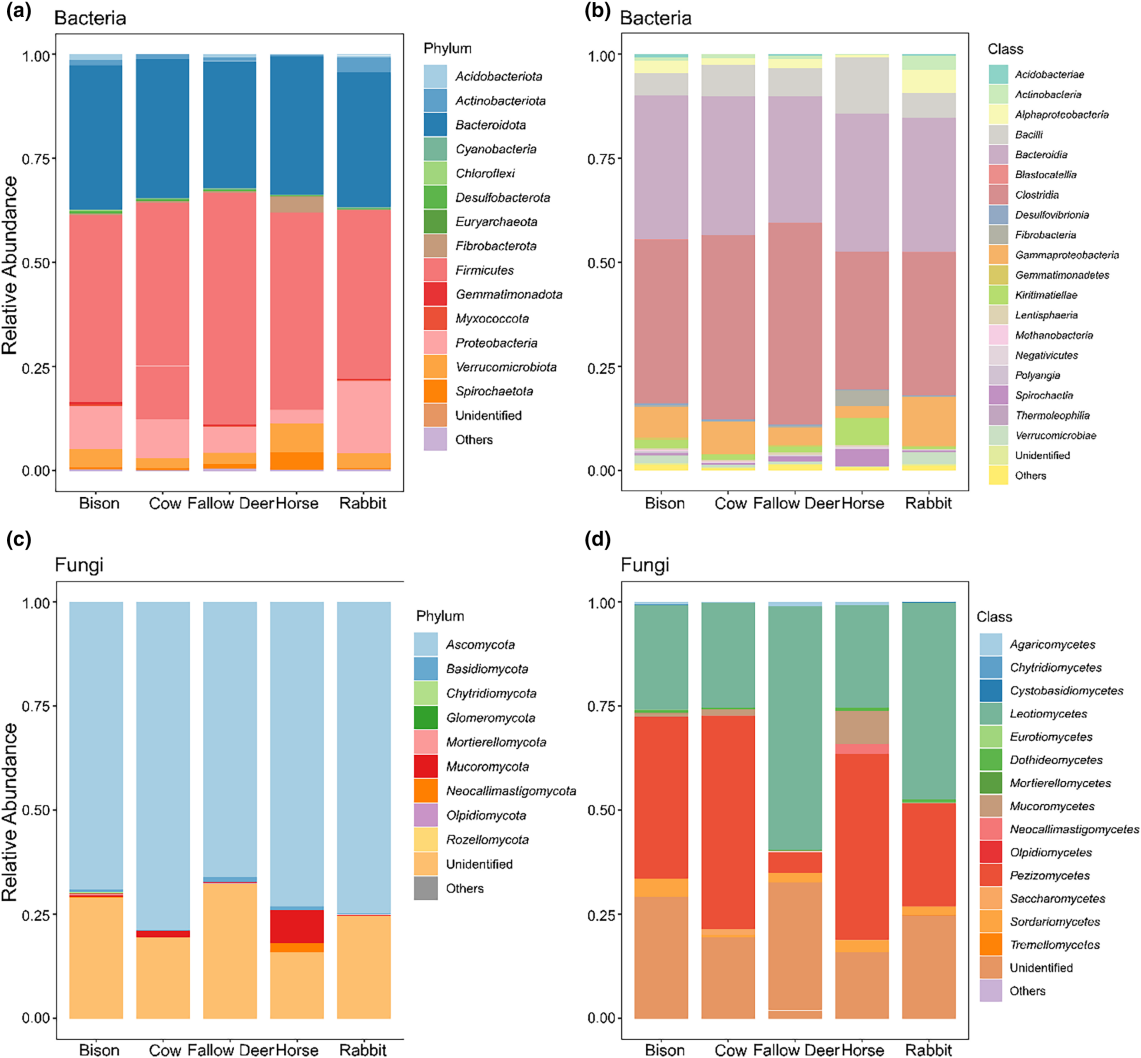
Taxonomic composition of bacterial and fungal communities in herbivore dung. (a) Relative abundance of the top 15 most abundant bacteria at the phylum level; (b) relative abundance of the top 20 most abundant bacteria at the class level; (c) relative abundance of the top 10 most abundant fungi at the phylum level; (d) relative abundance of the top 15 most abundant fungi at the class level. The comparison statistical results and exact *p*‐values of Kruskal–Wallis tests on relative abundance per microbe among five herbivore species are listed in Tables S2–S8, including those for bacterial genera.

At the phylum level, the dominant bacteria in the herbivore dung were *Firmicutes*, *Bacteroidota*, *Proteobacteria*, and *Verrucomicrobiota* (Figure 3a, Table S3). *Firmicutes* were the most abundant bacterial phyla and were particularly dominant (55.51%) in the dung of fallow deer. The abundance of *Bacteroidota* was the second most abundant bacterial phylum in the dung of the five herbivore species and did not show significant differences in abundance among species. The abundance of *Proteobacteria* (17.28%) was higher in rabbit dung than in the other dung types. In addition, *Verrucomicrobiota* (7.00%), *Fibrobacterota* (3.79%), and *Spirochaetota* (4.11%) were relatively abundant in horse dung. Further exploration of bacterial classes showed a relatively high abundance of *Bacilli*, *Kiritimatiellae*, *Fibrobacteria*, and *Spirochaetia* in horse dung, as well as a relatively high abundance of *Gammaproteobacteria* in rabbit dung (Figure 3b, Table S4). Additionally, we investigated the content of *Rhizobiales*, a bacterial order related to the nitrogen fixation of plants, in the dung bacteria community of the five herbivore species, which showed a relatively high abundance in the dung of bison and rabbit compared to the other three herbivore species (Figure S1).

*Ascomycota* was the most abundant fungal phylum, varying between 78.59% in cow dung and 65.94% in fallow deer dung (Figure 3c, Table S7). Horse dung had a relatively high abundance of *Mucoromycota* (7.92%) and *Neocallimastigomycota* (2.27%), which were significantly higher than their abundance in the other dung types. At the class level, the dung microbiota of the five herbivore species was dominated by *Leotiomycetes* and *Pezizomycetes* (Figure 3d, Table S8), which both belong to *Ascomycota*. *Leotiomycetes* was 58.32% in fallow deer dung, significantly higher than in the dung of the other herbivore species. The abundance of *Pezizomycetes* also varied considerably among the five herbivore species, with the highest abundance in cow (51.18%) and horse (44.74%), intermediate in European bison (38.74%) and rabbit (24.66%) and low in fallow deer dung (4.97%).

### 3.4 Functional characterization of the bacterial communities

**FIGURE 4:**
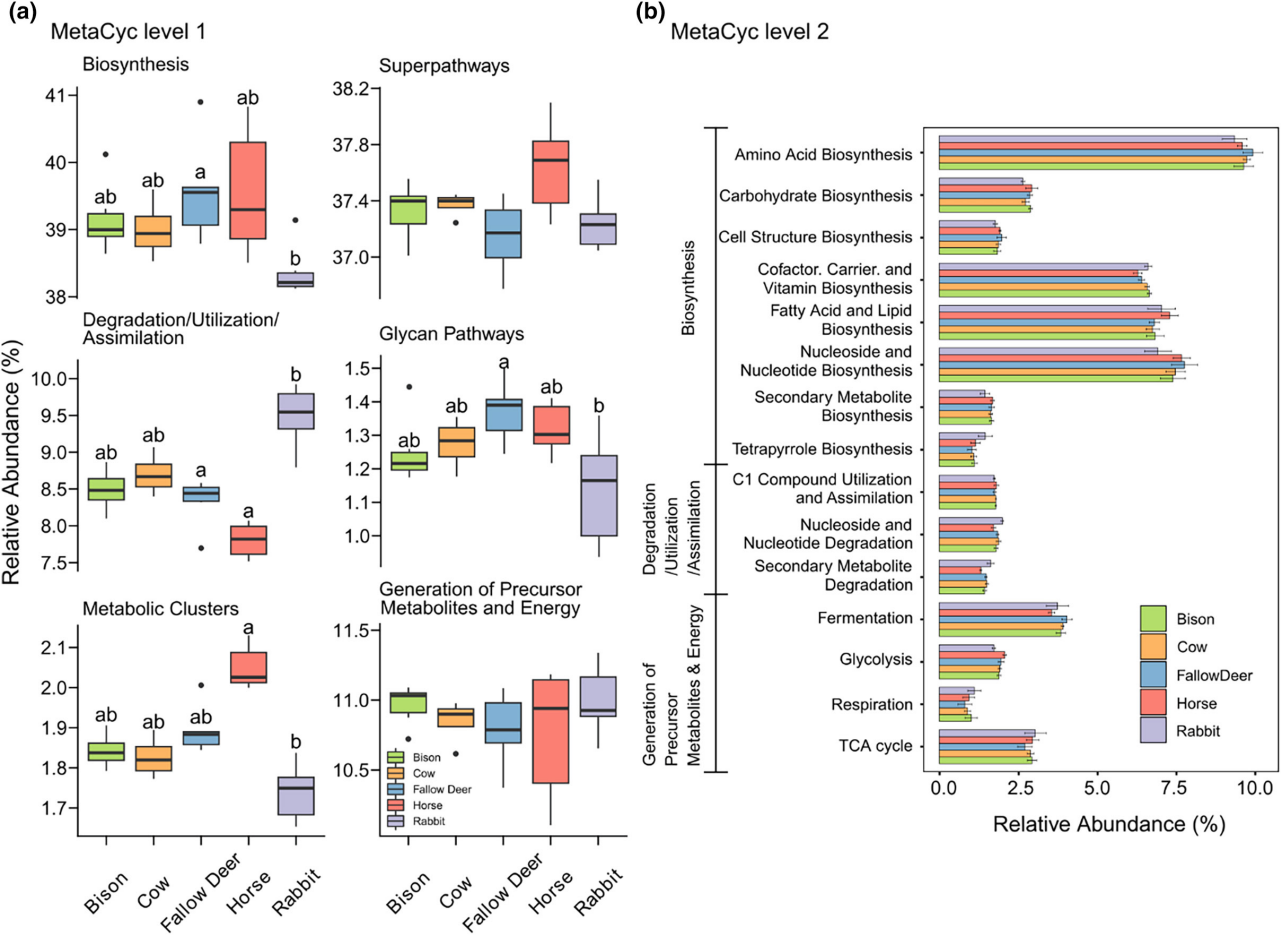
Functional characterization of the bacterial communities at the different levels of MetaCyc pathways using PICRUSt2 in the herbivore dung. (a) Relative abundance (>1%) at level 1; (b) Relative abundance (>1%) without unknown portion at level 2. Different letters indicate significant differences at the *p* < .05 level (*n* = 6); The exact *p*‐values are listed in Table S9, and the comparison statistical results at level 2 are listed in Table S11. The functional characteristics at MetaCyc level 3 and level 4 are presented in Figure S2, and their exact *p*‐values and comparison statistical results are listed in Tables S10, S12, and S13.

At level 1 of MetaCyc pathways, the bacterial communities of all herbivore dung exhibited mainly functional groups of “Biosynthesis,” “Super pathways,” “Generation of Precursor Metabolites and Energy,” “Degradation/Utilization/Assimilation,” “Metabolic Clusters,” and “Glycan Pathways” (Figure 4a). Rabbit dung deviated from the other dung types with the lower abundances of “Biosynthesis,” “Metabolic Clusters,” and “Glycan Pathways” as well as a higher abundance of “Degradation/Utilization/Assimilation” functional groups. Horse dung had a high abundance of “Metabolic Clusters” and a relatively low abundance of “Degradation/Utilization/Assimilation” functional groups. A further analysis of the functions of the bacterial community at level 2 of MetaCyc pathways showed that several functional subgroups with high relative abundance (>1%) were concentrated in “Biosynthesis,” “Degradation/Utilization/Assimilation,” and “Generation of Precursor Metabolites and Energy” (Figure 4b). At this level, except for the high abundance of rabbit dung in “Tetrapyrrole Biosynthesis,” rabbit dung with relatively low abundance in other functional subgroups of biosynthesis deviated from the dung of other four dung types. Additionally, rabbit dung had a significantly higher abundance in “Nucleoside Degradation” and “Secondary Metabolite Degradation” than other dung types, while the opposite was found in horse dung. Furthermore, horse dung had a low abundance of “Fermentation” and rabbit dung had a low abundance of “Glycolysis” (Figure 4b, Table S11). The additional detailed analyses at level 3 and level 4 of MetaCyc pathways (Figure S2, Tables S10, S12, and S13) further elaborated on the differences in the functional characteristics of bacterial communities in the dung of five herbivore species.

### 3.5 Functional characterization of the fungal communities

**FIGURE 5:**
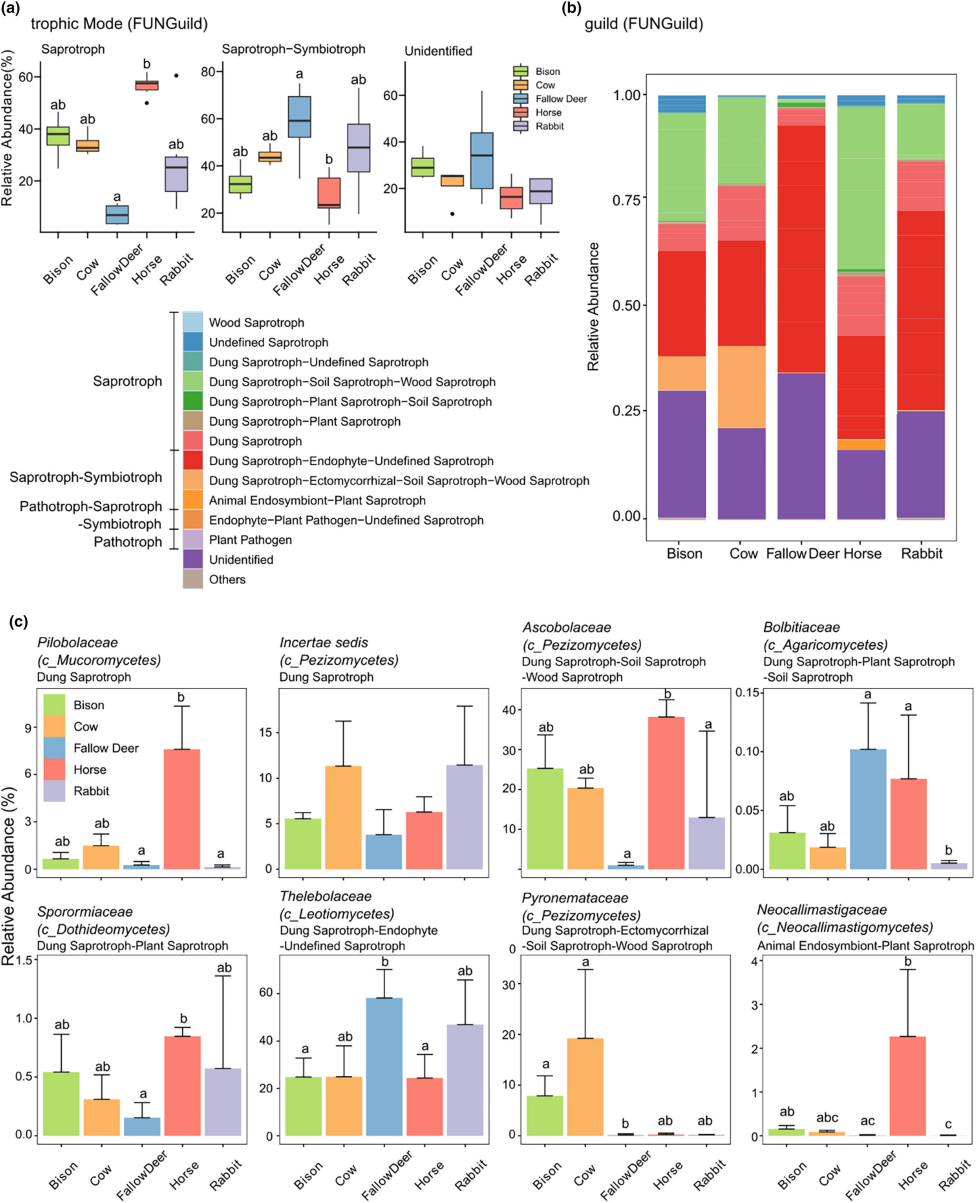
Functional characterization of the fungal communities at the different modes of FUNGuild in the herbivore dung. (a) Relative abundance (>1%) at the trophic Mode. (b) Compositions of predicted functional profiles at the guild mode; (c) Relative abundance of specific dominant fungal taxa at the family level corresponding to the functional groups at the guild mode (The first level title is the fungal species and the class where it is located; the secondary title is the functional groups corresponding to the fungi). Different letters indicate significant differences at the *p* < .05 level (*n* = 6); the comparison statistical results at the fungal guild mode are listed in Table S14; the exact *p*‐values are listed in Table S15.

First, the ASVs with fungal species annotations were analyzed in comparison with the FUNGuild database to obtain the distributions of functional groups at different modes. The dominant functional groups at the trophic mode were “Saprotroph” and “Saprotroph‐Symbiotroph,” and a relatively large portion of undefined trophic fungi (Figure 5a). There was a considerable difference in the abundances of “Saprotroph” and “Saprotroph‐Symbiotroph” between fallow deer dung and horse dung, that fallow deer dung had a relatively low abundance of “Saprotroph” and horse dung had a significantly high abundance of it, but this situation was reversed in the abundance of “Saprotroph‐Symbiotroph.”

Subsequently, we explored specific ecological guilds and further interpretative analysis of the fungal taxa associated with dominant functional subgroups. Saprotrophs were mainly composed of wood saprotroph, various dung saprotrophs and undefined saprotroph, and “Saprotroph‐Symbiotroph” was composed of various saprotrophs combined with endophyte, ectomycorrhizal, and animal endosymbiont (Figure 5b). The fungal taxa at the family level with the highest contribution of dung saprotroph were *Pilobolaceae* (belongs to class *Mucoromycota*) and *Incertae sedis* (belongs to class *Pezizomycetes*). The “dung saprotroph‐soil saprotroph‐wood saprotroph” subgroup was dominated by *Ascobolaceae* (belongs to class *Pezizomycetes*), except for fallow deer dung. The “dung saprotrophic‐endophyte‐undefined saprotroph” subgroup was dominated by *Thelebolaceae* (belongs to class *Leotiomycetes*), which showed a significantly high abundance in fallow deer dung. There were considerable differences in the abundance of the various fungal functional subgroups among the dung of five herbivore species (Figure 5c). For instance, horse diverged for *Pilobolaceae*, *Ascobolaceae*, and *Neocallimastigaceae* (an animal endosymbiont), cow and European bison diverged for *Pyronemataceae* (belongs to class *Pezizomycetes*), and fallow deer for *Thelebolaceae*.

### 3.6 The co‐occurrence network between the dung bacteria and fungi

**FIGURE 6:**
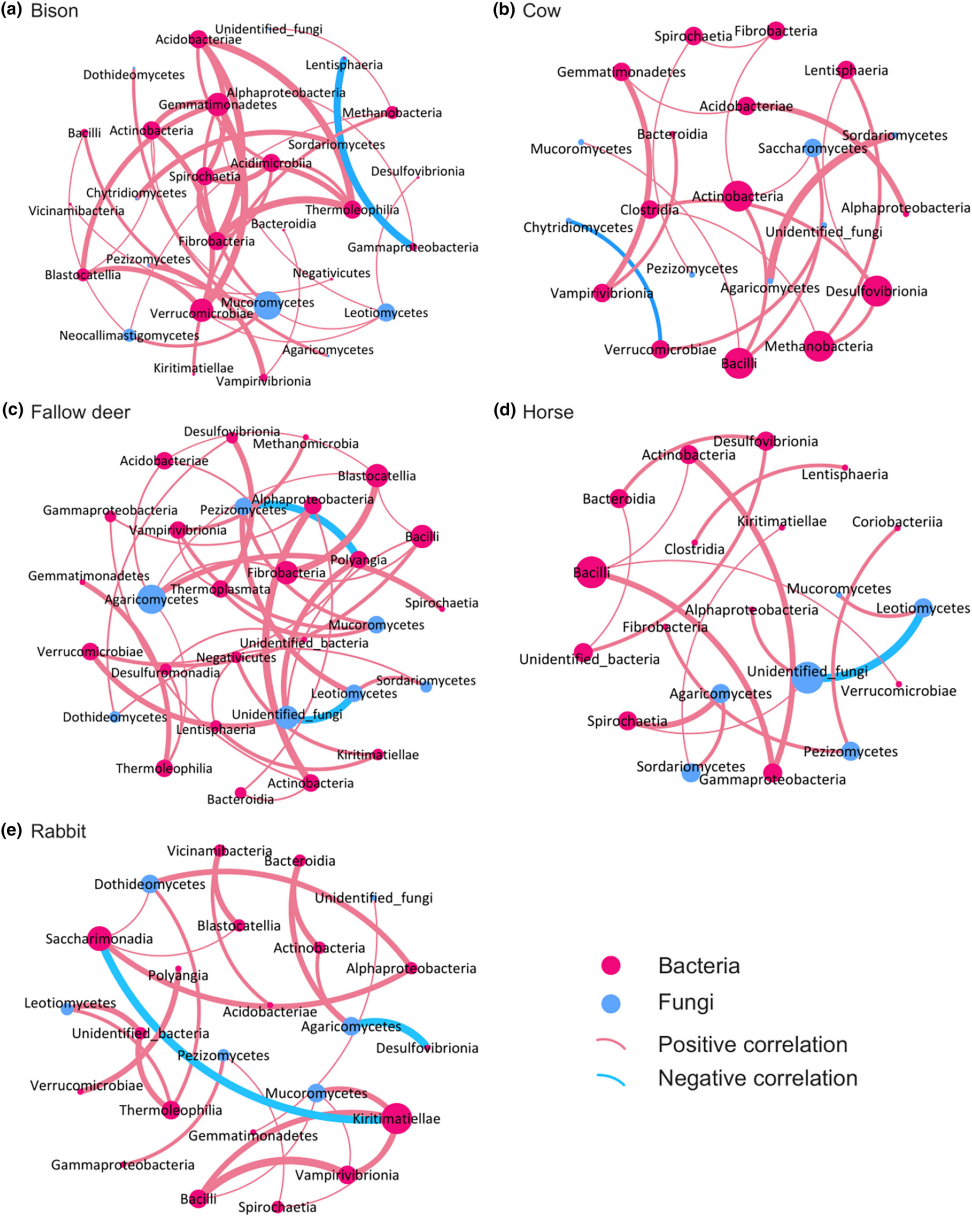
Co‐occurrence analysis of the bacterial and fungal community structure at the class level in the dung of European bison (a), cow (b), fallow deer (c), horse (d), and rabbit (e) inhabiting European coastal dunes.

We explored correlations between dung bacteria and fungi at the class level across the five herbivore species, constructing co‐occurrence networks (Figure 6). The networks exhibited varying complexity, with bison dung comprising 29 nodes and 36 edges, cow dung with 21 nodes and 19 edges, fallow deer dung with 29 nodes and 38 edges, horse dung with 20 nodes and 17 edges, and rabbit dung with 24 nodes and 26 edges.

Bison dung, with 68.97% bacteria and 31.03% fungi, revealed seven communities (modularity: 0.675, average clustering coefficient: 0.654), showcasing the pivotal roles of *Gemmatimonadetes*, *Verrucomicrobiae*, and *Mucoromycetes* sorted by degree (Figure 6a). Cow dung (66.67% bacteria, 33.33% fungi) exhibited five communities (modularity: 0.752, average clustering coefficient: 0.385), emphasizing *Bacilli* and *Actinobacteria* (Figure 6b). Fallow deer dung (75.86% bacteria, 24.14% fungi) displayed three communities (modularity: 0.672, average clustering coefficient: 0.465), with central roles played by *Agaricomycetes* and *Bacilli* (Figure 6c). Horse dung (70% bacteria, 30% fungi) featured five communities (modularity: 0.756, average clustering coefficient: 0.444), highlighting the crucial role of *Bacilli* (Figure 6d). Rabbit dung (75% bacteria, 25% fungi) presented four communities (modularity: 0.685, average clustering coefficient: 0.557), with key contributions from *Kiritimatiellae* (Figure 6e). Positive correlations between dung microbial classes in all five herbivore species exceeded 90%.

### 3.7 Microbial environmental factors analysis

**FIGURE 7:**
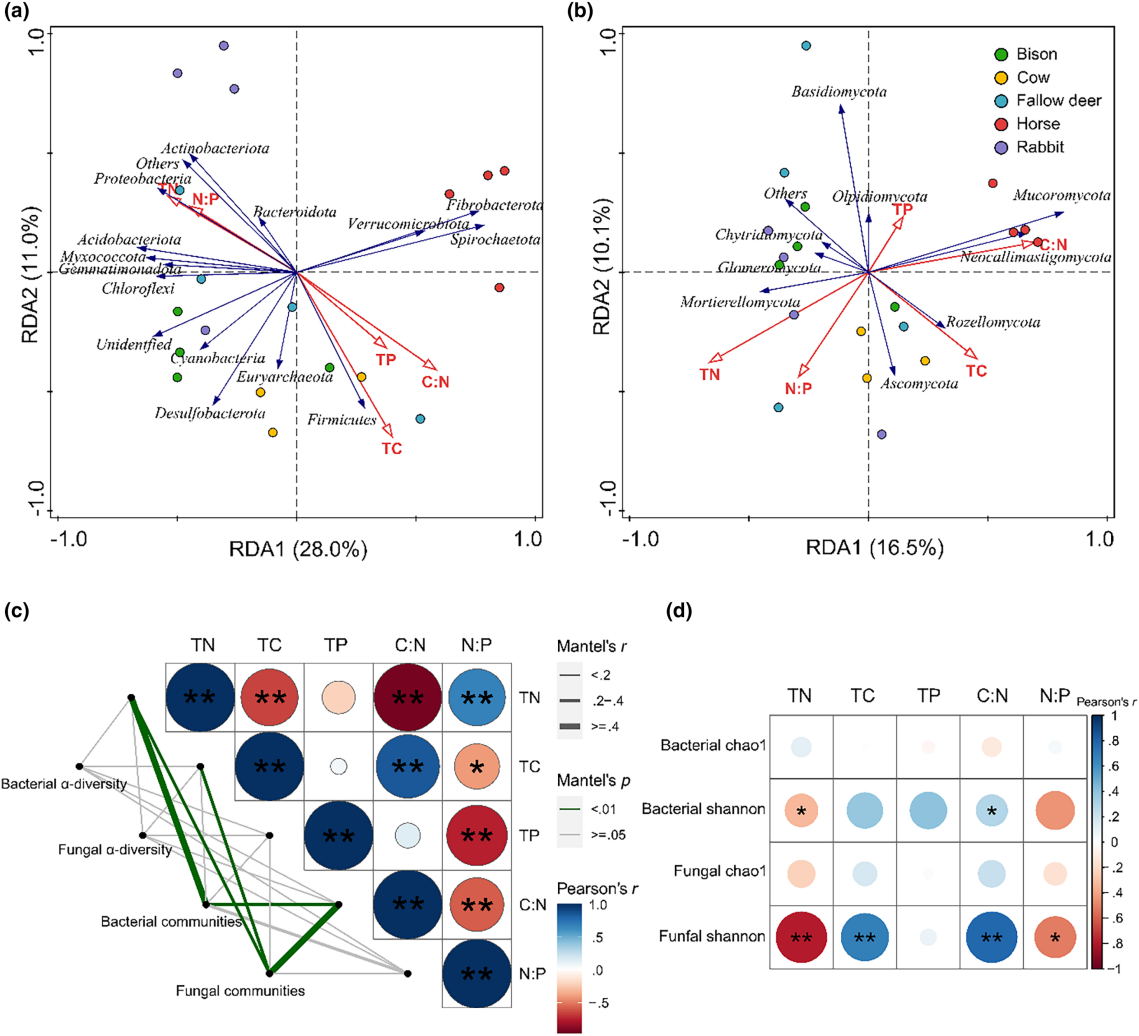
Correlations between dung C, N, P, and dung microbial community characteristics. Redundancy analysis (RDA) of dung bacterial (a) and fungal (b) community at the phyla level and dung physicochemical properties; (c) Mantel test evaluated correlations between dung microbial alpha diversity (based on Chao1 and Shannon indices) and dung community characteristics (based on the dominant abundance microbiome at the phyla level), and with dung C, N, and P; (d) Pearson correlation analysis between dung microbial alpha diversity indices and dung physicochemical properties; *.01 < *p* ≤ .05, **p ≤ .01; The results of Mantel test are listed in Table S16.

The results of the RDA analysis showed that the first and second axes, respectively, explained 28.0% and 11.0% of the variation in the bacterial phyla communities (Figure 7a). Dung C, C:N, and P were positively correlated with the relative abundance of *Verrucomicrobiota*, *Fibrobacterota*, *Spirochaetota*, and *Firmicutes*, whereas dung N and N:P were positively correlated with the relative abundances of *Actinobacteriota*, *Proteobacteria*, and *Bacteroidota*. The first two axes of the RDA for the fungal phyla communities accounted for 16.5% and 10.1% of the shifts, and dung N, C, C:N, and N:P were the prime environmental factors for variation in the fungal community structure (Figure 7b). The relative abundance of *Ascomycota* correlated positively with dung N, C, and N:P. Abundances of *Mucoromycota* and *Neocallimastigomycota* were positively correlated with the dung C and C:N.

The results of the Mantel test showed that dung N and C:N were the main environmental factors affecting the dung microbial community composition and α‐diversity (Figure 7c, Table S16). Furthermore, a Pearson correlation analysis showed that the diversity (Shannon–Wiener) of fungi and to a lesser extent also bacteria decreased with dung N and N:P in dung, whereas it increased with C and C:N. Dung P only correlated with bacterial diversity, not fungal diversity (Figure 7d).

## 4 DISCUSSION

### 4.1 Characterization of bacterial communities

Based on the number of total ASVs observed, bacteria are in definitive predominance representing about 88% to 90% of the microbial biomass in the dung of the five herbivore species, and that is generally consistent with previous studies about the intestinal or fecal microflora of herbivorous mammals (Newbold & Ramos‐Morales, 2020; Sun et al., 2020). The alpha diversity indices of the bacterial community in the dung revealed no significant differences in the richness or evenness among the three ruminants, while the non‐ruminant rabbit and horse dung showed significantly lower richness and evenness. Additionally, the beta diversity depicted by the NMDS representing interspecific differences also showed the difference in the bacterial community between ruminants and non‐ruminants. The separate projections of the two non‐ruminants were distinctly discriminated from overlapping those of the ruminants. Despite the convergence of diets due to the limited availability of forage in winter (Fu et al., 2021), this does not seem to exclude an effect of diet on differences in the bacterial diversity of the herbivore dung in this study. However, there are previous studies that found less correlation between differences in species, diet, and habitat and differences in microbial diversity (Budd et al., 2020; Kartzinel et al., 2019), with gut physiology being the most significant predictor of microbial diversity (Reese & Dunn, 2018). These putatively explained to some extent the differences in bacterial diversity in the dung of foregut ruminants (bison, cow, and fallow deer) and monogastric non‐ruminants with hindgut fermentation (horse and rabbit) in this study.

We found that the bacterial communities in the dung of the five herbivore species primarily consisted of *Firmicutes* and *Bacteroidota*, but that both *Proteobacteria* and *Verrucomicrobiota* were also relatively abundant. The phyla *Firmicutes* was most dominant in all five dung types, followed by *Bacteroidota*, both of which collectively constitute about 73% (rabbit) to 86% (fallow deer) of relative abundance. This is the general trend in numerous studies on the dung microbiota of herbivorous mammals such as cattle (*Aberdeen Angus*) (Durso et al., 2010), horse (*Equus csballus*) (Proudman et al., 2015), African savanna elephant (*Loxodonta Africana*) (Budd et al., 2020), and even the rodent Plateau zokor (*Eospalax baileyi*) (Liu et al., 2021). The predominance of *Firmicutes* and *Bacteroidota* represents the prominent capacity of hosts to degrade and utilize their fiber‐based diet (Gao et al., 2019; Girija et al., 2013; Sun et al., 2020). Furthermore, the ratio of *Firmicutes* to *Bacteroidota* was seemingly associated with seasonality, which potentially interacted with hosts' survival strategies (Dougal et al., 2017; Theelen et al., 2021). A study of the microbiota in horse dung found that *Firmicutes* had a significantly higher abundance in winter, while *Bacteroidota* had a significantly higher abundance in summer (Theelen et al., 2021), which is compatible with the season (late winter and early spring) in which our dung samples were collected. In addition, previous studies showed that significant decreases in Firmicutes and increases in Bacteroidota are associated with weight loss (Boshuizen et al., 2021; Dougal et al., 2017). To resist foraging scarcity under cold temperatures, herbivores consume more high‐fiber woody plants by debarking, etc. (Cromsigt et al., 2017; Saint‐Andrieux et al., 2009), and this requires the assistance of phylum *Firmicutes* including the classes *Clostridia* and *Bacilli*, which is known for its efficient degradation in complex plant organic materials, such as cellulose, hemicellulose, lignocellulose, and polysaccharide (Dowd et al., 2008; Flint, 2004; Gomez‐Flores et al., 2017; Murty & Chandra, 1991).

There were few significant differences in bacterial community composition between the dung of European bison, cow, and fallow deer, mainly due to the similar digestive system, especially the close phylogenetic relationship between bison and cows (Nilsson et al., 2012), and the convergence of diets (Table 1) because of the homogeneity of geographic environment and single seasonality potentially weakening the differences between their dung bacterial communities. Such bacterial community composition, in which Firmicutes and Bacteroidota dominate nearly 80% and Proteobacteria assist, is somewhat in line with the reported diet structure of these three ruminant species (Cromsigt et al., 2017; Merceron et al., 2014; Saint‐Andrieux et al., 2009). However, although the dietary preferences of horses were similar to those of bison, cow, and fallow deer (Valdés‐Correcher et al., 2018), we still found some differences in the composition of the dung bacterial community potentially driven by distinct digestive physiological factors or others. *Verrucomicrobiota*, *Fibrobacterota*, and *Spirochaetota* were relatively prominent in horse dung. The genus WCHB1‐41 of *Verrucomicrobiota* occupied a relatively higher abundance in the bacterial community of horse dung (Table S5), whose increase was shown to be associated with a low‐protein/high‐fiber diet of the herbivores at low temperatures (Guo et al., 2021). Whereas a fiber‐rich diet increases the thickness of the intestinal mucus (Desai et al., 2016), WCHB1‐41 plays an important role in the metabolic pathway degrading mucins and converting into arginine and fatty acids, providing energy to other members in the community and the host cells (Derrien et al., 2004; Guo et al., 2021). Additionally, the phylum *Spirochaetota* was found to be associated with the regulation of insulin sensitivity in horses, and its reduced abundance in the equine intestinal system is potentially correlated with insulin dysregulation (Boshuizen et al., 2021; Langner et al., 2020). Therefore, we speculate that such a bacterial community composition to some extent represented the relatively high disease resistance of horses inhabiting the National Park Zuid‐Kennemerland.

*Proteobacteria* accounted for a significantly higher proportion of the bacteria in rabbit dung than the dung of the other four herbivore species. The rabbit dung was collected in a coastal dune area with relatively low concealment but good food availability (Lombardi et al., 2003). One previous study found that there were interactions between concealment and dietary preference with plant secondary metabolites (PSMs), namely, rabbits would feed on more food with PSMs when the coverage is low (Utz et al., 2016). These presumably elucidated the higher abundance of *Proteobacteria* in rabbit dung, as the genus *Pseudomonas* (Table S5) of it was highly relevant to the biodegradation and detoxification of PSMs (Cipollone et al., 2008; Mekuto et al., 2016). In addition to the interaction with concealment, there was likely a linkage between the uptake of PSMs and a high‐fiber diet, with pygmy rabbits (*Brachylagus idahoensis*) living on shrubland having stronger tolerance to PSMs than other dietary generalist rabbits (Nobler et al., 2019). Furthermore, *Rhizobiales* within *Proteobacteria*, a bacterial order previously discovered in the dung of some mammalian herbivores and beetles with a low‐nitrogen, high‐cellulose diet (Martínez‐Romero et al., 2021; Suárez‐Moo et al., 2020), exhibited a relatively high abundance in rabbit dung (Figure S1). This bacterial order is known for its nitrogen‐fixing ability in plants, and in the gut of herbivory insects where their N_2_‐fixation also provides nitrogen to the herbivorous diet (Russell et al., 2009), but whether it plays the same role in the gut of rabbits or other mammalian herbivores remains to be confirmed. Generally, European wild rabbits prefer to forage low‐fiber herbaceous plants and feed on the roots or seeds of specific plant species, but benefiting from their coprophagous re‐digestive system (Ebino et al., 1993; Hirakawa, 2001), they also have the capacity of adaption to a variety of available food sources and maximization of the assimilation of nutrients (Funosas et al., 2021; Sheail et al., 1995; Van Strien et al., 2011). Such ecological plasticity enables them to rapidly establish their ecological niche in a range of habitats across spatial and seasonal dependencies.

Changes in the composition of bacterial communities corroborate with changes in their metabolic configurations (Boshuizen et al., 2021; Proudman et al., 2015). According to the database of experimentally elucidated metabolic pathways in MetaCyc, we predicted bacterial functions in the dung of five herbivore species, and found that bacterial functions were primarily expressed in “Biosynthesis,” “Super pathways,” “Generation of Precursor Metabolites and Energy,” and “Degradation/Utilization/Assimilation.” There were no significant differences in bacterial functional groups among the dung of European bison, cow, fallow deer, and horse, except for the lower abundance of rabbit dung on “Biosynthesis” and the higher abundance on “Degradation/Utilization/Assimilation.” The results of the secondary functional pathways of MetaCyc further indicated that the most dominant functional subgroups were primarily distributed in biosynthesis, degradation, and assimilation of nutrient metabolites, which were presumably compatible with the ecological effects and needs of bacteria in fresh dung. Moreover, the deviation of bacterial functions in rabbit dung may be related to its coprophagy and special detached colonic structure that enables them to rapidly excrete indigestible material (hard dung) while retaining some nutrients in the cecum for refermentation and reuse (Ebino et al., 1993; Hirakawa, 2001). Alternatively, these nutrients are excreted as “soft” dung, which is then immediately reconsumed (Ebino et al., 1993; Hirakawa, 2001). The rabbit dung we collected was their final metabolite product with bacteria more adapted to humus degradation and less to other biosynthetic procedures.

### 4.2 Characterization of fungal communities

Since bacteria are dominant whether in both gut microbiome or dung microbiome and fungi generally represent 10% to 20% of the microflora (Huws et al., 2018; Krause et al., 2013), most studies have focused on bacteria. While there is some debate about the contribution of fungi in microflora and less data on the characteristics of fungal communities in non‐human dung, the importance of fungi for metabolic activities and decomposition of excretion should not be ignored. In our study, the fungal diversity results showed that the alpha diversities of fungi in rabbit dung were slightly lower than in the dung of the other four herbivore species, and the beta diversity did not differ significantly among the five herbivore species. Furthermore, there were not many significant differences in the fungal community composition between the species. Compared to the fungal community structure of previous mammalian studies in which several fungal phyla collectively dominated (Sokol et al., 2017; Strati et al., 2016; Sun et al., 2018; Wheeler et al., 2016), our fungal communities were rather monotonous, with only Ascomycota phyla dominating (from 78.6% in cow dung to 65.9% in fallow deer dung), except for a small share of *Mucoromycota* and *Neocallimastigomycota* in horse dung. *Ascomycota* belongs to one of the most pervasive and diversified fungal phyla, they include many decomposers of organic matter and have been reported to dominate in soils amended with manure (Guo et al., 2018; Tayyab et al., 2019). At the class level, *Leotiomycetes* and *Pezizomycetes* were the dominant *Ascomycota* fungi classes in the dung of the five herbivore species. Interestingly, unlike in the dung of European bison, cow, and horse, where *Leotiomycetes* and *Pezizomycetes* were almost equally divided, *Leotiomycetes* was absolutely dominant in fallow deer dung while *Pezizomycetes* was only small fraction, as well as precariously dominating in rabbit dung. Subsequently, we found in the fungal functional prediction by FUNGuild that the genus *Thelebolus* as an endophyte contributed fully to the dominance of *Leotiomycetes*. *Thelebolus* is a psychrophilic ascomycetous fungus that was previously recorded for dung or the gut of thermostatic animals living in subarctic ecosystems, with some records on seabird dung and carcasses (De Hoog et al., 2005; Leotta et al., 2002). In contrast to the trend of *Thelebolaceae*, the family *Ascobolaceae* (belongs to *Pezizomycetes*) with a saprobiontic lifestyle was significantly scarce in fallow deer dung and less in rabbit dung, but more abundant in the dung of bison, cow, and horse, and particularly significantly highest in horse dung. *Ascobolaceae* are known as decomposers on humus and are represented as the “dung saprotroph‐soil saprotroph‐wood saprotroph” subgroup in FUNGuild, especially *Ascobolus michaudii* and *Ascobolus albidus* typically feeding on the dung of large herbivores and omnivorous mammals (Cannon & Kirk, 2007)which may be one factor for its low content in the dung of fallow deer and rabbit with smaller body size. Additionally, *Mucoromycota* (mostly *Pilobolus*), and *Neocallimastigomycota* had a slight presence in horse dung. The former is previously described as a coprophilous fungus playing a vital role in the decomposition of herbivore dung and nutrient recycling in ecosystems (Aluoch et al., 2017; Richardson, 2008), and the second is an anaerobic endosymbiont inhabiting the digestive tracts of larger mammalian herbivores with highly active cellulolytic and hemicellulolytic enzymes (Gruninger et al., 2014; Ljungdahl, 2008). Taken together, the fungi in horse dung were more biased toward saprotrophic mode than in fallow deer dung. In contrast, the fungi in fallow deer dung had more symbiotrophic properties in addition to the saprotrophic mode, and the fungal functions of the dung of bison, cow, and rabbit fell somewhere in between.

In addition, a co‐occurrence network analysis was conducted to explore interactions among bacterial and fungal communities across the dung of five herbivore species (Figure 6). The most complicated networks were observed in bison and fallow deer dung in terms of their nodes and edges, while the most stable community structure was identified in cow dung based on modularity and average clustering coefficient. This aligns with the findings from the analysis of bacterial and fungal community diversity indices, revealing that the three ruminant dung microbial communities with higher richness and evenness concurrently exhibited more complex and stable microbial community structures. Moreover, the predominantly positive correlations among bacterial and fungal classes indicate a high synergy within intestinal environments, involving the co‐propagation or execution of metabolic functions (Wunderlich et al., 2023), as well as a symbiotic relationship between bacteria and fungi within the dung of the five herbivore species. Nevertheless, the inherent connections between bacteria and fungi in herbivore dung warrant further exploration.

### 4.3 Microbial communities and nutrients

We observed significant correlations between microbial communities and nutrient concentrations and/or ratios in the dung of herbivores. The abundance of *Firmicutes* was significantly positively correlated with P, C, and C:N, while the abundance of *Proteobacteria* and *Bacteroidota* were positively correlated with N and N:P. The dominant fungal flora, *Ascomycota* was positively correlated with C and N:P. These observations on the correlation between the abundance of these microbial species and environmental factors in the herbivore dungare in line with the results of previous studies about the variation of the microbial community in the soil fertilized by dung (Iqbal et al., 2022; Tayyab et al., 2019). The diversity of both fungi and bacteria increased with C and C:N in our study, which is in line with studies about the variation of soil microbial community relating to its stoichiometry (Yang et al., 2022; Zhu et al., 2022). We also found that fungal and bacterial diversity decreased with dung N and N:P, which agrees with the results of the meta‐analysis for the effects of N addition on soil bacterial diversity (Wang et al., 2023; Zhang & Han, 2012) but those studies did not find an effect of N on fungal diversity. Overall, our results illustrate that a relatively high N and low C in the dung stimulates dominance and lower diversity of these decomposing microbes, and predict the decisive effect on the variation of soil microbial community when the dung is deposited into the soil.

Sitters et al. (2014) showed that rates of herbivore dung deposition and nutrient return were driven by the dung C:N:P stoichiometry. Transposed to the herbivore dung types analyzed in the present study, this would imply that, for example, rabbit dung with a higher N concentration and higher N:P would release more and faster N into the soil than the other four dung types. Furthermore, variation in dung C:N:P among herbivore species is affected by diet and body size (Le Roux et al., 2020; Sitters & Olde Venterink, 2021b), where the example of the rabbit probably has a relatively high dung N:P ratio partly because it is has a low body weight. Our present study indicates that the relatively high abundance of *Proteobacteria*, particularly *Rhizobiales*, in rabbit dung, may also play a role in contributing to nitrogen cycling and absorbing nutrients from the herbivory diet in the gut, even perhaps keeping the activity of promoting N_2_‐fixating after dung excretion. So far, this remains speculative and merits further study, but a high dung N:P stoichiometry of, for instance, rabbit dung has a significant impact on plant species competition as demonstrated in our previous experiments (Sitters & Olde Venterink, 2021a; Valdés‐Correcher et al., 2019). Using rabbit dung as an example, we provide a potentially new perspective to further explain how herbivore dung affects important ecosystem processes such as decomposition, nutrient cycling, and plant species competition, and the role of the dung microbial composition in this merits further study.

## AUTHOR CONTRIBUTIONS

**Xingzhao Sun:** Conceptualization (lead); data curation (lead); formal analysis (lead); methodology (equal); project administration (equal); validation (equal); visualization (lead); writing – original draft (lead). **Judith Sitters:** Conceptualization (equal); funding acquisition (equal); project administration (equal); resources (equal); supervision (equal); writing – review and editing (equal). **Joske Ruytinx:** Conceptualization (equal); data curation (supporting); methodology (supporting); resources (supporting); validation (supporting); writing – review and editing (supporting). **Martin J. Wassen:** Methodology (supporting); resources (equal); supervision (supporting); validation (equal); writing – review and editing (supporting). **Harry Olde Venterink:** Conceptualization (equal); funding acquisition (equal); methodology (supporting); project administration (equal); resources (lead); supervision (lead); validation (equal); writing – review and editing (lead).

## Supporting information


Data S1.

## Data Availability

Data have been archived on publicly accessible repositories: 16s rRNA and ITS sequences available from NCBI Sequence Read Archive with BioProject accession Nos. PRJNA853949; Dung nutrient data available from the Dryad Digital Repository https://doi.org/10.5061/dryad.x95x69pqn.
