# Correction to “Microbial community composition in the dung of five sympatric European herbivore species”

**DOI:** 10.1002/ece3.11601

**Published:** 2024-06-19

Sun, X., Sitters, J., Ruytinx, J., Wassen, M. J., & Olde Venterink, H. (2024). Microbial community composition in the dung of five sympatric European herbivore species. *Ecology and Evolution*, 14(3), e11071. https://doi.org/10.1002/ece3.11071

In Table 2, the positions of decimal points of TC, TN, and TP were incorrect. The corrected Table 2 reads as follows:

**TABLE 2:** Moisture and nutrient concentrations in the dung of five herbivore species collected in the dune area of the National Park Zuid Kennemerland, the Netherlands in February 2020.

Herbivore species	Moisture (%)	TC (g kg^−1^)	TN (g kg^−1^)	TP (g kg^−1^)	C:N	N:P
Bison	81.15 ± 0.94^a^	482 ± 5^ab^	13.9 ± 0.2^bc^	2.24 ± 0.08^ab^	34.82 ± 0.87^b^	6.21 ± 0.22^b^
Cow	81.90 ± 1.43^a^	516 ± 3^a^	13.3 ± 0.1^bc^	1.91 ± 0.04^b^	38.84 ± 0.46^ab^	6.97 ± 0.19^b^
Fallow Deer	69.08 ± 0.95^c^	446 ± 9^cd^	15.6 ± 0.8^b^	3.12 ± 0.29^a^	28.81 ± 0.90^c^	5.12 ± 0.42^b^
Horse	76.15 ± 0.93^ab^	478 ± 4^bc^	12.1 ± 0.1^c^	2.44 ± 0.23^ab^	39.39 ± 0.75^a^	5.14 ± 0.40^b^
Rabbit	70.28 ± 1.22^bc^	421 ± 5^d^	18.2 ± 0.1^a^	1.48 ± 0.14^b^	23.21 ± 0.27^d^	12.87 ± 1.59^a^

*Note*: Mean ± Standard error of the mean (SEM) (*n* = 4); Different superscript letters indicate significant differences between species using one‐way ANOVA followed by Scheffe test (*p* < .05).

We apologize for this error.

