# Supplementary material for: Microbial community composition in the dung of five sympatric European herbivore species
Source: Ecol Evol. 2024 Mar 13;14(3):e11071. doi: 10.1002/ece3.11071 (PMC10933625; doi:10.1002/ece3.11071)
Supplement: Supplementary file 1 — Data S1. [file ECE3-14-e11071-s001.docx]

Table S1. *P*-values of bacterial and fungal alpha diversity indices calculated by one-way Kruskal-Wallis test (supplement to Fig. 2A & 2B)

| Indices | Bacteria | Fungi |
| --- | --- | --- |
| Chao1 | 0.292 | 0.071 |
| Pielou evenness | <0.001 | 0.003 |
| Shannon | <0.001 | 0.003 |
| Simpson | <0.001 | 0.002 |

Table S2. *P*-values of the relative abundance of the dung bacterial communities at the phylum (top15), class (top20) and genus (top20) levels calculated by one-way Kruskal-Wallis test (supplement to Fig. 3A & 3B)

| Bacteria | | | | | |
| --- | --- | --- | --- | --- | --- |
| phylum | *P*-value | class | *P*-value | genus | *P*-value |
| Firmicutes | 0.017 | Clostridia | 0.003 | UCG.010 | <0.001 |
| Bacteroidota | 0.422 | Bacteroidia | 0.399 | Rikenellaceae_RC9_gut_group | 0.236 |
| Proteobacteria | 0.001 | Gammaproteobacteria | 0.001 | UCG.005 | <0.001 |
| Verrucomicrobiota | 0.008 | Bacilli | 0.002 | Bacteroides | 0.009 |
| Acidobacteriota | 0.011 | Alphaproteobacteria | 0.002 | Alistipes | 0.001 |
| Actinobacteriota | 0.001 | Kiritimatiellae | <0.001 | Pedobacter | 0.001 |
| Gemmatimonadota | 0.017 | Verrucomicrobiae | 0.001 | Clostridia_vadinBB60_group | 0.015 |
| Desulfobacterota | <0.001 | Acidobacteriae | 0.011 | WCHB1.41 | <0.001 |
| Myxococcota | 0.015 | Actinobacteria | 0.001 | Eubacterium_coprostanoligenes_group | 0.001 |
| Euryarchaeota | 0.001 | Gemmatimonadetes | 0.014 | Prevotellaceae_UCG.004 | <0.001 |
| Fibrobacterota | 0.002 | Desulfovibrionia | <0.001 | Clostridia_UCG.014 | 0.004 |
| Spirochaetota | <0.001 | Polyangia | 0.003 | p.251.o5 | 0.001 |
| Unidentified | 0.002 | Lentisphaeria | <0.001 | Pseudomonas | 0.002 |
| Cyanobacteria | 0.365 | Blastocatellia | 0.005 | RF39 | 0.006 |
| Chloroflexi | 0.019 | Methanobacteria | 0.001 | Bacteroidales_RF16_group | 0.002 |
| Others | 0.066 | Fibrobacteria | 0.002 | Christensenellaceae_R.7_group | 0.003 |
|  |  | Spirochaetia | <0.001 | Ruminococcus | 0.011 |
|  |  | Unidentified | 0.003 | Flavobacterium | 0.003 |
|  |  | Thermoleophilia | 0.020 | Treponema | <0.001 |
|  |  | Negativicutes | <0.001 | Unidentified | 0.166 |
|  |  | Others | 0.007 | Others | 0.003 |

Table S3. Statistical results with significant letters of the relative abundance (%) of dominant bacterial phyla top 15 (supplement to Fig. 3A)

| Bacterial phyla | Bison | Cow | Fallow deer | Horse | Rabbit |
| --- | --- | --- | --- | --- | --- |
| *Firmicutes* | 44.77±2.31a | 52±0.7ab | 55.51±2.72b | 47.24±1.41ab | 40.35±5.04ab |
| *Bacteroidota* | 34.54±0.8a | 33.35±1.52a | 30.27±1.54a | 33.15±1.77a | 32.31±2.54a |
| *Proteobact-eria* | 10.33±1.49ab | 9.28±1.54ab | 6.34±1.21a | 3.26±0.62a | 17.28±2.03b |
| *Verrucomic-robiota* | 4.37±0.55ab | 2.33±0.16a | 2.58±0.29a | 7±0.54b | 3.61±1.1ab |
| *Acidobacte-riota* | 1.45±0.29a | 0.02±0ab | 0.72±0.23ab | 0.11±0.1b | 0.82±0.17ab |
| *Actinobact-eriota* | 1.23±0.11ab | 1.16±0.09ab | 1.09±0.34ab | 0.38±0.06a | 3.56±1.32b |
| *Gemmatim-onadota* | 0.6±0.12a | 0.03±0ab | 0.27±0.09ab | 0.06±0.05b | 0.35±0.07ab |
| *Desulfobac-terota* | 0.55±0.04a | 0.41±0.04abc | 0.45±0.03ab | 0.32±0.01bc | 0.25±0.02c |
| *Myxococco-ta* | 0.42±0.08a | 0.01±0b | 0.17±0.05ab | 0.06±0.04ab | 0.24±0.05ab |
| *Euryarchae-ota* | 0.16±0.06a | 0.1±0.05a | 0.01±0ab | 0.01±0.01ab | 0±0b |
| *Fibrobacterota* | 0.31±0.1ab | 0.33±0.07ab | 0.43±0.1ab | 3.79±0.57a | 0.14±0.05b |
| *Spirochaet-ota* | 0.34±0.06ab | 0.38±0.07abc | 1.15±0.1ac | 4.11±0.43c | 0.17±0.07b |
| *Cyanobacte-ria* | 0.29±0.05a | 0.23±0.03a | 0.32±0.06a | 0.16±0.01a | 0.3±0.1a |
| *Chloroflexi* | 0.08±0.02a | 0±0a | 0.04±0.01a | 0.01±0a | 0.04±0.01a |
| *Unidentified* | 0.16±0.01a | 0.22±0.03a | 0.14±0.01ab | 0.09±0.01b | 0.17±0.01a |
| *Others* | 0.4±0.08a | 0.15±0.02a | 0.52±0.05a | 0.25±0.08a | 0.42±0.1a |

Note: Mean ± Standard error of the mean (SEM) (n=6); Different letters indicate significant differences (Kruskal- Wallis test followed by Dunn’s pairwise comparison test, p < 0.05).

Table S4. Statistical results with significant letters of the relative abundance (%) of dominant bacterial classes top 20 (supplement to Fig. 3B)

| Bacterial classes | Bison | Cow | Fallow deer | Horse | Rabbit |
| --- | --- | --- | --- | --- | --- |
| *Clostridia* | 39.19±2.36ab | 44.17±0.7ab | 48.4±2.69a | 33.11±1.28b | 34.37±4.38ab |
| *Bacteroidia* | 34.49±0.8a | 33.35±1.52a | 30.24±1.54a | 33.15±1.77a | 32.26±2.54a |
| *Gammaproteo-bacteria* | 7.41±1.14ab | 7.77±1.55ab | 4.21±0.72a | 2.75±0.5a | 11.67±1.28b |
| *Bacilli* | 5.29±0.35a | 7.52±0.58ab | 6.8±0.42ab | 13.54±2b | 5.86±0.75a |
| *Alphaproteo-bacteria* | 2.93±0.37a | 1.51±0.06ab | 2.14±0.59ab | 0.51±0.16b | 5.61±1.65a |
| *Kiritimatiellae* | 2.02±0.45ab | 1.34±0.1ab | 1.42±0.23ab | 6.52±0.57a | 0.53±0.15b |
| *Verrucomicro-biae* | 1.87±0.22a | 0.62±0.13ab | 0.54±0.07ab | 0.14±0.06b | 2.88±1.07a |
| *Acidobacteriae* | 0.9±0.18a | 0.01±0ab | 0.46±0.15ab | 0.07±0.07b | 0.54±0.11ab |
| *Actinobacteria* | 0.8±0.04ab | 1.09±0.1a | 0.86±0.3ab | 0.23±0.03b | 3.29±1.31a |
| *Gemmatimona-detes* | 0.55±0.11a | 0.03±0ab | 0.25±0.08ab | 0.06±0.05b | 0.31±0.06ab |
| *Desulfovibrio-nia* | 0.48±0.04a | 0.38±0.03ab | 0.3±0.02ab | 0.21±0.01b | 0.2±0.01b |
| *Polyangia* | 0.35±0.07a | 0±0b | 0.14±0.05ab | 0.04±0.04b | 0.21±0.04ab |
| *Lentisphaeria* | 0.46±0.04a | 0.37±0.02ab | 0.61±0.06a | 0.33±0.04ab | 0.18±0.02b |
| *Blastocatellia* | 0.27±0.06a | 0±0b | 0.12±0.04ab | 0.02±0.02b | 0.11±0.03ab |
| *Methanobacte-ria* | 0.16±0.06a | 0.1±0.05a | 0.01±0ab | 0.01±0.01ab | 0±0b |
| *Fibrobacteria* | 0.31±0.1ab | 0.33±0.07ab | 0.43±0.1ab | 3.79±0.57a | 0.14±0.05b |
| *Spirochaetia* | 0.34±0.06ab | 0.38±0.07abc | 1.15±0.1ac | 4.11±0.43c | 0.17±0.07b |
| *Thermoleophi-lia* | 0.23±0.05a | 0±0ab | 0.1±0.04ab | 0.02±0.02b | 0.12±0.03ab |
| *Negativicutes* | 0.21±0.04ab | 0.25±0.05ab | 0.25±0.04ab | 0.51±0.04a | 0.08±0.02b |
| *Unidentified* | 0.28±0.01a | 0.28±0.03a | 0.22±0.01ab | 0.17±0.01b | 0.23±0.02ab |
| *Others* | 1.46±0.25a | 0.49±0.06b | 1.37±0.18ab | 0.72±0.09ab | 1.26±0.08ab |

Note: Mean ± SEM (n=6); Different letters indicate significant differences (Kruskal- Wallis test followed by Dunn’s pairwise comparison test, p < 0.05).

Table S5. Statistical results with significant letters of the relative abundance (%) of dominant bacterial genera top 20 (supplement to Fig. 3A & 3B)

| Bacterial genera | Bison | Cow | Fallow deer | Horse | Rabbit |
| --- | --- | --- | --- | --- | --- |
| *UCG.010* | 10.4±1.02ab | 9.2±0.23abc | 13.86±1.83a | 6.82±0.72bc | 4.92±0.48c |
| *Rikenellaceae_RC9 _gut_group* | 6.82±0.06a | 6.61±0.22a | 6.99±0.35a | 6.65±0.55a | 5.25±1.35a |
| *UCG.005* | 7.81±0.21ab | 7.5±0.12abc | 8.96±0.38a | 3.88±0.47bc | 3.08±0.29c |
| *Bacteroides* | 4.35±0.27a | 3.91±0.27ab | 2.97±0.3ab | 1.35±0.24b | 2.77±1.07ab |
| *Alistipes* | 3.6±0.08a | 3.74±0.17a | 3.62±0.2a | 1.8±0.23b | 2.05±0.25ab |
| *Pedobacter* | 0.83±0.19a | 2.37±0.02ab | 1.5±0.53ab | 0.33±0.16a | 8.2±2.79b |
| *Clostridia_vadinBB60 _group* | 1.98±0.3ab | 2.81±0.08ab | 2.49±0.29ab | 1.48±0.12a | 3.57±0.58b |
| *WCHB1.41* | 2.02±0.45ab | 1.34±0.1ab | 1.43±0.23ab | 6.51±0.57a | 0.53±0.15b |
| *Eubacterium_copro-stanoligenes_group* | 2.59±0.06ab | 2.94±0.13a | 2.51±0.15ab | 1.65±0.14b | 1.81±0.23b |
| *Prevotellaceae_UCG. 004* | 3.51±0.43a | 2.81±0.47a | 2.62±0.34a | 1.27±0.23ab | 0.57±0.06b |
| *Clostridia_UCG.014* | 0.95±0.29a | 2.6±0.14ab | 1.67±0.42ab | 0.94±0.17a | 4.44±0.95b |
| *p.251.o5* | 0.67±0.18a | 0.99±0.16ab | 1.25±0.2ab | 6.45±0.46b | 0.39±0.2a |
| *Pseudomonas* | 1.12±0.14a | 2.07±0.38ab | 1.52±0.29ab | 0.79±0.12a | 3.28±0.58b |
| *RF39* | 1.01±0.19a | 1.62±0.04ab | 1.51±0.21ab | 0.89±0.11a | 2.65±0.52b |
| *Bacteroidales_RF16 _group* | 1.69±0.18a | 1.46±0.08ab | 1.35±0.27ab | 2.04±0.17a | 0.5±0.08b |
| *Christensenellaceae _R.7_group* | 1.44±0.05ab | 1.41±0.08ab | 1.7±0.12a | 1.48±0.06ab | 0.88±0.1b |
| *Ruminococcus* | 0.4±0.13a | 1.21±0.05ab | 1.15±0.16ab | 1.5±0.2b | 2.16±0.49b |
| *Flavobacterium* | 0.66±0.1ab | 0.89±0.08ab | 0.63±0.23ab | 0.16±0.04a | 3.63±1.48b |
| *Treponema* | 0.3±0.05ab | 0.33±0.06abc | 1.06±0.08ac | 3.82±0.42c | 0.15±0.06b |
| Unidentified | 21.67±0.78a | 21.06±1.02a | 20.07±0.64a | 20.9±0.94a | 19.08±0.52a |
| Others | 26.2±1.66ab | 23.15±0.39ab | 21.16±1.48a | 29.33±0.91b | 30.09±1.66b |

Note: Mean ± SEM (n=6); Different letters indicate significant differences (Kruskal- Wallis test followed by Dunn’s pairwise comparison test, p < 0.05).

Table S6. *P*-values of the relative abundance of the dung fungal communities at the phylum (top 10) and class (top15) levels calculated by one-way Kruskal-Wallis test (supplement to Fig. 3C & 3D)

| Fungi | | | |
| --- | --- | --- | --- |
| phylum | *P*-value | class | *P*-value |
| Ascomycota | 0.284 | Leotiomycetes | 0.007 |
| Basidiomycota | 0.003 | Pezizomycetes | 0.002 |
| Chytridiomycota | <0.001 | Unidentified | 0.135 |
| Glomeromycota | 0.039 | Sordariomycetes | 0.040 |
| Mortierellomycota | 0.196 | Mucoromycetes | <0.001 |
| Mucoromycota | <0.001 | Agaricomycetes | <0.001 |
| Neocallimastigomycota | <0.001 | Dothideomycetes | 0.017 |
| Olpidiomycota | 0.125 | Neocallimastigomycetes | <0.001 |
| Rozellomycota | 0.726 | Saccharomycetes | 0.061 |
| Unidentified | 0.135 | Cystobasidiomycetes | 0.142 |
| Others | 0.085 | Eurotiomycetes | 0.003 |
|  |  | Chytridiomycetes | 0.001 |
|  |  | Mortierellomycetes | 0.196 |
|  |  | Tremellomycetes | 0.010 |
|  |  | Olpidiomycetes | 0.125 |
|  |  | Others | 0.003 |

Table S7. Statistical results with significant letters of the relative abundance (%) of dominant fungal phyla top 10 (supplement to Fig. 3C)

| Fungal phyla | Bison | Cow | Fallow deer | Horse | Rabbit |
| --- | --- | --- | --- | --- | --- |
| *Ascomycota* | 68.97±2.34a | 78.59±3.4a | 65.94±8.18a | 73.05±2.17a | 74.72±9.63a |
| *Basidiomycota* | 0.65±0.09ab | 0.29±0.06a | 1.23±0.32b | 0.87±0.24ab | 0.3±0.15a |
| *Chytridiomycota* | 0.33±0.15a | 0.02±0.01ab | 0±0b | 0±0b | 0±0b |
| *Glomeromycota* | 0.01±0a | 0±0a | 0±0a | 0±0a | 0±0a |
| *Mortierellomycota* | 0.01±0.01a | 0±0a | 0.01±0.01a | 0±0a | 0.02±0.01a |
| *Mucoromycota* | 0.83±0.25ab | 1.51±0.37ab | 0.28±0.09a | 7.92±1.48b | 0.24±0.09a |
| *Neocallimastigo-mycota* | 0.15±0.03ab | 0.09±0.02abc | 0.01±0.01ac | 2.27±0.68b | 0.01±0c |
| *Olpidiomycota* | 0.01±0a | 0±0a | 0.01±0a | 0±0a | 0.01±0a |
| *Rozellomycota* | 0±0a | 0±0a | 0±0a | 0±0a | 0±0a |
| Unidentified | 29.04±2.18a | 19.51±3.76a | 32.51±8.21a | 15.88±2.9a | 24.71±9.66a |
| Others | 0±0a | 0±0a | 0±0a | 0±0a | 0±0a |

Note: Mean ± SEM (n=6); Different letters indicate significant differences (Kruskal- Wallis test followed by Dunn’s pairwise comparison test, p < 0.05).

Table S8. Statistical results with significant letters of the relative abundance (%) of dominant fungal classes top 15 (supplement to Fig. 3D)

| Fungal classes | Bison | Cow | Fallow deer | Horse | Rabbit |
| --- | --- | --- | --- | --- | --- |
| *Leotiomycetes* | 25.12±3.28a | 25.14±6.62ab | 58.32±5.97b | 24.44±4.06a | 47.06±7.71ab |
| *Pezizomycetes* | 38.74±4.52ab | 51.18±8.23a | 4.97±1.4b | 44.74±2.13a | 24.66±7.59ab |
| *Sordariomycetes* | 4.29±1a | 0.65±0.19b | 2.27±1.05ab | 2.82±0.38ab | 2.12±0.39ab |
| *Mucoromycetes* | 0.83±0.25ab | 1.51±0.37ab | 0.28±0.09a | 7.92±1.48b | 0.24±0.09a |
| *Agaricomycetes* | 0.6±0.1ab | 0.26±0.05ab | 1.11±0.24a | 0.86±0.25a | 0.12±0.03b |
| *Dothideomycetes* | 0.69±0.2ab | 0.32±0.1ab | 0.25±0.05a | 0.88±0.03b | 0.71±0.43ab |
| *Neocallimastigo-mycetes* | 0.15±0.03ab | 0.09±0.02abc | 0.01±0.01ac | 2.27±0.68b | 0.01±0c |
| *Saccharomycetes* | 0.04±0.03a | 1.29±1.22a | 0.03±0.01a | 0.13±0.05a | 0.05±0.01a |
| *Cystobasidiomy-cetes* | 0.04±0.01a | 0.02±0.01a | 0.01±0a | 0.01±0a | 0.15±0.13a |
| *Eurotiomycetes* | 0.07±0.01a | 0±0b | 0.02±0.01ab | 0.02±0.01ab | 0.06±0.01a |
| *Chytridiomycetes* | 0.12±0.08a | 0.01±0.01a | 0±0a | 0±0a | 0±0a |
| *Mortierellomycetes* | 0.01±0.01a | 0±0a | 0.01±0.01a | 0±0a | 0.02±0.01a |
| *Tremellomycetes* | 0.01±0ab | 0±0a | 0.01±0ab | 0±0ab | 0.02±0.01b |
| *Olpidiomycetes* | 0.01±0a | 0±0a | 0.01±0a | 0±0a | 0.01±0a |
| Unidentified | 29.26±2.15a | 19.53±3.76a | 32.69±8.19a | 15.89±2.9a | 24.78±9.65a |
| Others | 0.01±0a | 0±0ab | 0±0ab | 0±0b | 0.01±0ab |

Note: Mean ± SEM (n=6); Different letters indicate significant differences (Kruskal- Wallis test followed by Dunn’s pairwise comparison test, p < 0.05).

Table S9. *P*-values of the relative abundance of the dung bacterial functional groups at level 1 and level 2 (>1%) of MetaCyc pathways calculated by one-way Kruskal-Wallis test (supplement to Fig. 4)

| MetaCyc pathway | | | |
| --- | --- | --- | --- |
| Level 1 | *P*-value | Level 2 (>1%) | *P*-value |
| Biosynthesis | 0.034 | Amino Acid Biosynthesis | 0.045 |
| Degradation/Utilization/Assimilation | <0.001 | Carbohydrate Biosynthesis | 0.003 |
| Generation of Precursor Metabolites and Energy | 0.716 | Cell Structure Biosynthesis | 0.008 |
| Glycan Pathways | 0.040 | Cofactor. Carrier. and Vitamin Biosynthesis | 0.001 |
| Metabolic Clusters | <0.001 | Fatty Acid and Lipid Biosynthesis | 0.030 |
| Superpathways | 0.070 | Nucleoside and Nucleotide Biosynthesis | 0.016 |
|  |  | Secondary Metabolite Biosynthesis | 0.015 |
|  |  | Tetrapyrrole Biosynthesis | 0.009 |
|  |  | C1 Compound Utilization and Assimilation | 0.022 |
|  |  | Nucleoside and Nucleotide Degradation | <0.001 |
|  |  | Secondary Metabolite Degradation | <0.001 |
|  |  | Fermentation | 0.010 |
|  |  | Glycolysis | <0.001 |
|  |  | Respiration | 0.115 |
|  |  | TCA cycle | 0.207 |

Table S10. *P*-values of the relative abundance of the dung bacterial functional groups at level 3 (>1%) and level 4 (>1%) of MetaCyc pathways calculated by one-way Kruskal-Wallis test (supplement to Fig. 4)

| MetaCyc pathway | | | |
| --- | --- | --- | --- |
| Level 3 (>1%) | *P*-value | Level 4 (>1%) | *P*-value |
| Proteinogenic Amino Acid Biosynthesis | 0.037 | Purine Nucleotide De Novo Biosynthesis | 0.047 |
| Purine Nucleotide Biosynthesis | 0.019 | L-isoleucine Biosynthesis | 0.024 |
| Fatty Acid Biosynthesis | 0.024 | Unsaturated Fatty Acid Biosynthesis | 0.051 |
| Sugar Biosynthesis | 0.003 | Sugar Nucleotide Biosynthesis | 0.002 |
| Vitamin Biosynthesis | 0.001 | L-lysine Biosynthesis | 0.012 |
| Fermentation to Short-Chain Fatty Acids | 0.016 | Autotrophic CO2 Fixation | 0.001 |
| Carrier Biosynthesis | 0.001 | Folate Biosynthesis | 0.001 |
| Cell Wall Biosynthesis | 0.008 | CDP-diacylglycerol Biosynthesis | 0.080 |
| Purine Nucleotide Degradation | <0.001 | L-arginine Biosynthesis | 0.101 |
| Enzyme Cofactor Biosynthesis | 0.099 | 5-Aminoimidazole Ribonucleotide Biosynthesis | 0.007 |
| CO2 Fixation | 0.001 | UDP-N-Acetylmuramoyl-Pentapeptide Biosynthesis | 0.018 |
| Pyrimidine Nucleotide Biosynthesis | 0.003 | Hemiterpene Biosynthesis | 0.056 |
| Phospholipid Biosynthesis | 0.056 | Coenzyme A Biosynthesis | 0.007 |
| Sugar Derivative Degradation | <0.001 | Pyrimidine Nucleotide De Novo Biosynthesis | 0.009 |
| Terpenoid Biosynthesis | 0.056 | NAD Metabolism | 0.014 |
| Aerobic Respiration | 0.126 |  |  |

Table S11. Statistical results with significant letters of the relative abundance (%) of the bacterial functional groups at level 2 (>1%) of MetaCyc pathways (supplement to Fig. 4B)

| MetaCyc subpathways | Bison | Cow | Fallow deer | Horse | Rabbit |
| --- | --- | --- | --- | --- | --- |
| Amino Acid Biosynthesis | 9.65±0.13ab | 9.75±0.05ab | 9.94±0.13a | 9.61±0.06ab | 9.37±0.16b |
| Carbohydrate Biosynthesis | 2.9±0.02a | 2.74±0.05ab | 2.88±0.03a | 2.93±0.08a | 2.66±0.02b |
| Cell Structure Biosynthesis | 1.84±0.05ab | 1.88±0.03ab | 1.98±0.06a | 1.93±0.01a | 1.78±0.03b |
| Cofactor. Carrier. and Vitamin Biosynthesis | 6.66±0.03a | 6.59±0.03abc | 6.41±0.04bc | 6.3±0.05b | 6.63±0.04ac |
| Fatty Acid and Lipid Biosynthesis | 6.85±0.11a | 6.77±0.1a | 6.82±0.07a | 7.31±0.11a | 7.05±0.18a |
| Nucleoside and Nucleotide Biosynthesis | 7.41±0.16ab | 7.49±0.15ab | 7.78±0.17a | 7.69±0.11a | 6.93±0.17b |
| Secondary Metabolite Biosynthesis | 1.66±0.03ab | 1.63±0.03ab | 1.67±0.04ab | 1.69±0.02a | 1.45±0.06b |
| Tetrapyrrole Biosynthesis | 1.11±0.04ab | 1.09±0.04ab | 1.03±0.06a | 1.15±0.06ab | 1.46±0.09b |
| C1 Compound Utilization and Assimilation | 1.8±0.01a | 1.79±0.01a | 1.76±0.02a | 1.82±0.03a | 1.74±0.01a |
| Nucleoside and Nucleotide Degradation | 1.8±0.02a | 1.88±0.04ab | 1.85±0.02ab | 1.72±0.03a | 2±0.02b |
| Secondary Metabolite Degradation | 1.44±0.02ab | 1.51±0.02ab | 1.49±0.01ab | 1.32±0.01a | 1.63±0.04b |
| Fermentation | 3.85±0.06ab | 3.92±0.02ab | 4.05±0.06a | 3.56±0.04b | 3.75±0.15ab |
| Glycolysis | 1.9±0.02ab | 1.92±0.02ab | 1.96±0.04a | 2.07±0.02a | 1.73±0.02b |
| Respiration | 1.02±0.08a | 0.9±0.05a | 0.81±0.09a | 0.94±0.08a | 1.11±0.08a |
| TCA cycle | 2.94±0.06a | 2.89±0.05a | 2.71±0.09a | 2.96±0.08a | 3.05±0.14a |

Note: Mean ± SEM (n=6); Different letters indicate significant differences (Kruskal- Wallis test followed by Dunn’s pairwise comparison test, p < 0.05).

Table S12. Statistical results with significant letters of the relative abundance (%) of the bacterial functional groups at level 3 (>1%) of MetaCyc pathways (supplement to Fig. 4)

| MetaCyc subpathways | Bison | Cow | Fallow deer | Horse | Rabbit |
| --- | --- | --- | --- | --- | --- |
| Proteinogenic Amino Acid Biosynthesis | 9.13±0.12ab | 9.23±0.05ab | 9.4±0.12a | 9.08±0.06ab | 8.86±0.15b |
| Purine Nucleotide Biosynthesis | 5.71±0.13ab | 5.8±0.12ab | 6.02±0.13a | 5.9±0.08ab | 5.38±0.14b |
| Fatty Acid Biosynthesis | 4.95±0.13a | 4.9±0.11a | 4.92±0.08a | 5.42±0.08a | 5.24±0.19a |
| Sugar Biosynthesis | 2.9±0.02a | 2.74±0.05ab | 2.88±0.03a | 2.93±0.08a | 2.66±0.02b |
| Vitamin Biosynthesis | 2.83±0.01a | 2.79±0.04ab | 2.69±0.03b | 2.85±0.04a | 2.69±0.01b |
| Fermentation to Short-Chain Fatty Acids | 2.17±0.04ab | 2.2±0.02ab | 2.28±0.04a | 2.04±0.02b | 2.16±0.08ab |
| Carrier Biosynthesis | 2.03±0.05a | 1.93±0.04ab | 1.86±0.05ab | 1.72±0.02b | 2.18±0.08a |
| Cell Wall Biosynthesis | 1.84±0.05ab | 1.88±0.03ab | 1.98±0.06a | 1.93±0.01a | 1.78±0.03b |
| Purine Nucleotide Degradation | 1.78±0.02a | 1.85±0.03ab | 1.82±0.02ab | 1.66±0.02a | 1.98±0.02b |
| Enzyme Cofactor Biosynthesis | 1.8±0.03a | 1.87±0.03a | 1.85±0.04a | 1.73±0.03a | 1.75±0.04a |
| CO2 Fixation | 1.7±0.01a | 1.66±0ab | 1.64±0.01ab | 1.62±0.01b | 1.61±0.01b |
| Pyrimidine Nucleotide Biosynthesis | 1.7±0.03ab | 1.69±0.04ab | 1.76±0.04a | 1.79±0.03a | 1.55±0.03b |
| Phospholipid Biosynthesis | 1.51±0.02ab | 1.53±0ab | 1.57±0.03a | 1.53±0.01ab | 1.47±0.03b |
| Sugar Derivative Degradation | 1.44±0.02ab | 1.51±0.02ab | 1.49±0.01ab | 1.32±0.01a | 1.63±0.04b |
| Terpenoid Biosynthesis | 1.28±0.03ab | 1.28±0.03ab | 1.34±0.04a | 1.3±0.02ab | 1.13±0.05b |
| Aerobic Respiration | 0.99±0.08a | 0.88±0.05a | 0.79±0.08a | 0.92±0.08a | 1.07±0.08a |

Note: Mean ± SEM (n=6); Different letters indicate significant differences (Kruskal- Wallis test followed by Dunn’s pairwise comparison test, p < 0.05).

Table S13. Statistical results with significant letters of the relative abundance (%) of the bacterial functional groups at level 4 (>1%) of MetaCyc pathways (supplement to Fig. 4)

| MetaCyc subpathways | Bison | Cow | Fallow deer | Horse | Rabbit |
| --- | --- | --- | --- | --- | --- |
| Purine Nucleotide De Novo Biosynthesis | 3.61±0.08ab | 3.7±0.07ab | 3.81±0.08a | 3.68±0.05ab | 3.49±0.08b |
| L-isoleucine Biosynthesis | 3.31±0.05a | 3.33±0.03a | 3.41±0.05a | 3.17±0.05a | 3.18±0.06a |
| Unsaturated Fatty Acid Biosynthesis | 2.94±0.04a | 2.95±0.04a | 3.01±0.03a | 3.23±0.07a | 3.06±0.1a |
| Sugar Nucleotide Biosynthesis | 2.3±0.02a | 2.13±0.04ab | 2.25±0.02ab | 2.3±0.07a | 2.08±0.02b |
| L-lysine Biosynthesis | 2.1±0.04a | 2.16±0.02a | 2.23±0.05a | 2.23±0.01a | 2.03±0.06a |
| Autotrophic CO2 Fixation | 1.7±0.01a | 1.66±0ab | 1.64±0.01ab | 1.62±0.01b | 1.61±0.01b |
| Folate Biosynthesis | 1.53±0.01ab | 1.53±0.02ab | 1.5±0.02a | 1.62±0.02b | 1.48±0.01a |
| CDP-diacylglycerol Biosynthesis | 1.51±0.02a | 1.53±0a | 1.56±0.03a | 1.52±0.01a | 1.47±0.03a |
| L-arginine Biosynthesis | 1.49±0.02a | 1.5±0.01a | 1.52±0.02a | 1.5±0.01a | 1.43±0.03a |
| 5-Aminoimidazole Ribonucleotide Biosynthesis | 1.48±0.03ab | 1.46±0.03ab | 1.53±0.03a | 1.54±0.02a | 1.35±0.03b |
| UDP-N-Acetylmuramoyl-Pentapeptide Biosynthesis | 1.36±0.03ab | 1.37±0.03ab | 1.43±0.04a | 1.41±0.03a | 1.24±0.04b |
| Hemiterpene Biosynthesis | 1.28±0.03ab | 1.28±0.03ab | 1.34±0.04a | 1.3±0.02ab | 1.13±0.05b |
| Coenzyme A Biosynthesis | 1.24±0.02ab | 1.23±0.02ab | 1.26±0.02a | 1.23±0.02ab | 1.1±0.02b |
| Pyrimidine Nucleotide De Novo Biosynthesis | 1.22±0.02ab | 1.22±0.02ab | 1.26±0.03a | 1.27±0.02a | 1.11±0.03b |
| NAD Metabolism | 1.05±0.01a | 1.04±0.02ab | 1.04±0.02ab | 0.99±0.03ab | 0.95±0.02b |

Note: Mean ± SEM (n=6); Different letters indicate significant differences (Kruskal- Wallis test followed by Dunn’s pairwise comparison test, p < 0.05).

Table S14. Statistical results with significant letters of the relative abundance (%) of the fungal functional groups at the guild mode of FUNGuild (supplement to Fig. 5B)

| Guild mode | Bison | Cow | Fallow deer | Horse | Rabbit |
| --- | --- | --- | --- | --- | --- |
| Wood Saprotroph | 0.06±0.04a | 0±0a | 0±0a | 0±0a | 0±0a |
| Undefined Saprotroph | 4.03±0.98a | 0.44±0.08b | 0.83±0.21b | 2.45±0.34ab | 1.93±0.36ab |
| Dung Saprotroph-Undefined Saprotroph | 0.17±0.16ab | 0.02±0.01ab | 0.01±0a | 0.32±0.13b | 0.1±0.05ab |
| Dung Saprotroph-Soil Saprotroph-Wood Saprotroph | 25.28±3.43ab | 20.34±1.25ab | 0.98±0.31a | 38.16±2.09b | 12.96±8.85a |
| Dung Saprotroph-Plant Saprotroph-Soil Saprotroph | 0.31±0.09ab | 0.19±0.06ab | 1.02±0.18a | 0.77±0.22a | 0.05±0.01b |
| Dung Saprotroph-Plant Saprotroph | 0.56±0.14ab | 0.33±0.12ab | 0.18±0.07a | 0.95±0.05b | 0.58±0.37ab |
| Dung Saprotroph | 6.25±0.3ab | 12.84±2.8ab | 4.07±1.2a | 13.92±1.14b | 11.59±3.04ab |
| Dung Saprotroph-Endophyte-Undefined Saprotroph | 24.81±3.27a | 24.92±6.55ab | 58.17±6.01b | 24.4±4.06a | 46.95±7.7ab |
| Dung Saprotroph-Ectomycorrhizal-Soil Saprotroph-Wood Saprotroph | 7.88±1.61a | 19.22±7.42a | 0.17±0.1b | 0.27±0.1ab | 0.2±0.03ab |
| Animal Endosymbiont-Plant Saprotroph | 0.15±0.03ab | 0.09±0.02abc | 0.01±0.01ac | 2.27±0.68b | 0.01±0c |
| Plant Pathogen | 0.05±0.02a | 0±0b | 0.03±0.02ab | 0.01±0ab | 0.01±0ab |
| Endophyte-Plant Pathogen-Undefined Saprotroph | 0.02±0.02a | 0±0a | 0±0a | 0±0a | 0±0a |
| Unidentified | 29.83±2.24a | 21.4±4.1a | 34.3±7.49a | 16.28±2.9a | 25.12±9.66a |
| Others | 0.6±0.09a | 0.2±0.07ab | 0.22±0.05b | 0.19±0.05b | 0.5±0.24ab |

Note: Mean ± SEM (n=6); Different letters indicate significant differences (Kruskal- Wallis test followed by Dunn’s pairwise comparison test, p < 0.05).

Table S15. *P*-values of the relative abundance of the dung fungal functional groups at trophic mode, guild mode of FUNGuild, as well as that of specific dominant fungal families corresponding to the functional groups at the guild mode calculated by one-way Kruskal-Wallis test (supplement to Fig. 5)

| FunGuild | | | | | |
| --- | --- | --- | --- | --- | --- |
| trophicMode | *P*-value | guildMode | *P*-value | specific family associated with guild mode | *P*-value |
| Saprotroph-Symbiotroph | 0.007 | Wood Saprotroph | 0.090 | Thelebolaceae | 0.007 |
| Saprotroph | <0.001 | Undefined Saprotroph | 0.001 | Pyronemataceae | 0.001 |
| Unidentified | 0.101 | Dung Saprotroph-Undefined Saprotroph | 0.044 | Neocallimastigaceae | <0.001 |
| Others | 0.052 | Dung Saprotroph-Soil Saprotroph-Wood Saprotroph | 0.001 | Ascobolaceae | 0.001 |
|  |  | Dung Saprotroph-Plant Saprotroph-Soil Saprotroph | <0.001 | Bolbitiaceae | <0.001 |
|  |  | Dung Saprotroph-Plant Saprotroph | 0.012 | Sporormiaceae | 0.010 |
|  |  | Dung Saprotroph | 0.011 | Incertae sedis | 0.144 |
|  |  | Dung Saprotroph-Endophyte-Undefined Saprotroph | 0.007 | Pilobolaceae | <0.001 |
|  |  | Dung Saprotroph-Ectomycorrhizal-Soil Saprotroph-Wood Saprotroph | 0.001 |  |  |
|  |  | Animal Endosymbiont-Plant Saprotroph | <0.001 |  |  |
|  |  | Plant Pathogen | 0.008 |  |  |
|  |  | Endophyte-Plant Pathogen-Undefined Saprotroph | 0.101 |  |  |
|  |  | Unidentified | 0.101 |  |  |
|  |  | Others | 0.011 |  |  |

Table S16. The results of the Mantel test in the correlation between the dung physicochemical properties and dung microbial community characteristics (supplement to Fig. 7C)

| dung microbial alpha-diversity & community composition | dung physicochemical properties | R-value | *P*-value | rd | pd |
| --- | --- | --- | --- | --- | --- |
| Bacterial alpha diversity | TN | 0.00327 | 0.411 | <0.2 | >=0.05 |
|  | TC | -0.08064 | 0.763 | <0.2 | >=0.05 |
|  | TP | -0.08574 | 0.68 | <0.2 | >=0.05 |
|  | C:N | -0.01505 | 0.532 | <0.2 | >=0.05 |
|  | N:P | -0.07555 | 0.645 | <0.2 | >=0.05 |
| Fungal alpha diversity | TN | -0.11918 | 0.909 | <0.2 | >=0.05 |
|  | TC | 0.010485 | 0.46 | <0.2 | >=0.05 |
|  | TP | 0.005311 | 0.386 | <0.2 | >=0.05 |
|  | C:N | -0.08117 | 0.813 | <0.2 | >=0.05 |
|  | N:P | -0.126 | 0.838 | <0.2 | >=0.05 |
| Bacterial community composition | TN | 0.445725 | 0.002 | >=0.4 | <0.01 |
|  | TC | 0.087204 | 0.24 | <0.2 | >=0.05 |
|  | TP | 0.121701 | 0.227 | <0.2 | >=0.05 |
|  | C:N | 0.369055 | 0.001 | 0.2-0.4 | <0.01 |
|  | N:P | 0.260687 | 0.103 | 0.2-0.4 | >=0.05 |
| Fungal community composition | TN | 0.371719 | 0.002 | 0.2-0.4 | <0.01 |
|  | TC | 0.361876 | 0.001 | 0.2-0.4 | <0.01 |
|  | TP | 0.1371 | 0.108 | <0.2 | >=0.05 |
|  | C:N | 0.477005 | 0.002 | >=0.4 | <0.01 |
|  | N:P | 0.088995 | 0.199 | <0.2 | >=0.05 |


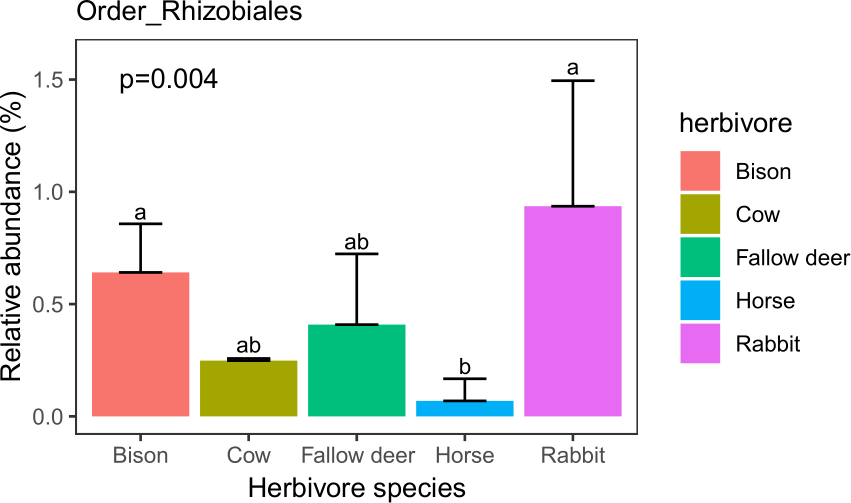


Figure S1. Relative abundance of *Rhizobiales* (one bacterial order encompassing symbiotic nitrogen-fixing species, including beneficial root-nodule bacteria) in the dung of five herbivore species. Different letters indicate significant differences (*P*<0.05 by one-way Kruskal-Wallis test, n=6) among five herbivore species.





Figure S2. Functional characterization of the dung bacterial communities at the deeper levels of MetaCyc pathways using PICRUSt2 (supplement to Fig. 4). A Relative abundance (>1%) at level 3; B Relative abundance (>1%) at level 4. The full statistical results with exact *P*-values at level 3 and level 4 are listed in Tables S10, S12 and S13.
